# Defective chromatin architectures in embryonic stem cells derived from somatic cell nuclear transfer impair their differentiation potentials

**DOI:** 10.1038/s41419-021-04384-2

**Published:** 2021-11-16

**Authors:** Dan-Ya Wu, Xinxin Li, Qiao-Ran Sun, Cheng-Li Dou, Tian Xu, Hainan He, Han Luo, Haitao Fu, Guo-Wei Bu, Bingbing Luo, Xia Zhang, Bin-Guang Ma, Cheng Peng, Yi-Liang Miao

**Affiliations:** 1 Institute of Stem Cell and Regenerative Biology, College of Animal Science and Veterinary Medicine, Huazhong Agricultural University, 430070 Wuhan, Hubei China; 2 Key Laboratory of Agricultural Animal Genetics, Breeding and Reproduction (Huazhong Agricultural University), Ministry of Education, 430070 Wuhan, Hubei China; 3 Hubei Key Laboratory of Agricultural Bioinformatics, College of Informatics, Huazhong Agricultural University, 430070 Wuhan, Hubei China; 4 Center for Life Sciences, School of Life Sciences, Yunnan University, 650500 Kunming, China; 5 Hubei Hongshan Laboratory, 430070 Wuhan, China

**Keywords:** Embryonic stem cells, Reprogramming, Stem-cell differentiation

## Abstract

Nuclear transfer embryonic stem cells (ntESCs) hold enormous promise for individual-specific regenerative medicine. However, the chromatin states of ntESCs remain poorly characterized. In this study, we employed ATAC-seq and Hi-C techniques to explore the chromatin accessibility and three-dimensional (3D) genome organization of ntESCs. The results show that the chromatin accessibility and genome structures of somatic cells are re-arranged to ESC-like states overall in ntESCs, including compartments, topologically associating domains (TADs) and chromatin loops. However, compared to fertilized ESCs (fESCs), ntESCs show some abnormal openness and structures that have not been reprogrammed completely, which impair the differentiation potential of ntESCs. The histone modification H3K9me3 may be involved in abnormal structures in ntESCs, including incorrect compartment switches and incomplete TAD rebuilding. Moreover, ntESCs and iPSCs show high similarity in 3D genome structures, while a few differences are detected due to different somatic cell origins and reprogramming mechanisms. Through systematic analyses, our study provides a global view of chromatin accessibility and 3D genome organization in ntESCs, which can further facilitate the understanding of the similarities and differences between ntESCs and fESCs.

## Introduction

Nuclear transfer embryonic stem cells (ntESCs) provide a resource of patient-matched cells for personalized therapies and disease models with less teratogenic, which hold enormous promise for regenerative medicine [1, 2]. Both fertilized ESCs (fESCs) and ntESCs have similar developmental potentials, such as teratoma formation and full-term development of tetraploid complemented embryos [3–5]. Previous studies revealed that ntESCs showed more abnormities compared to fESCs, including mismatched mitochondria [6], abnormal DNA methylation patterns [7] and others [2, 8, 9]. Moreover, cloned mice suffer from more diseases, such as obesity, organ dysplasia and short lifetimes [10–13], which suggest that the existence of abnormities impedes the development of post-implantation cloned embryos and the growth of cloned animals. In addition to ntESCs, induced pluripotent stem cells (iPSCs) are another pluripotent stem cells derived by somatic cell reprogramming and provide potential medical applications [14, 15]. They have unique differences in their genetic and epigenetic [16, 17].

Recently, we and another group showed that abnormal 3D genome structures were present in SCNT embryos, including aberrant enhancer-promoter interactions of ZGA genes, stronger TAD boundaries and others [18, 19]. Whether these defects can be passed on to ntESCs remains unclear. In addition, it is reported that the reprogramming process of iPSCs can erase the somatic-cell-specific genome structures and establish an ESC-like 3D genome [20, 21]. However, the chromatin accessibility and 3D genome of ntESCs remain poorly understood, and there are no comparisons between ntESCs and iPSCs in 3D genome organization. In this study, we compared ntESCs with fESCs as well as iPSCs to explore the chromatin accessibility and 3D genome changes during reprogramming.

## Materials and methods

### Animals and nuclear transfer

Mice were housed in a temperature-controlled environment under a 12 h light: 12 h dark cycle. All animal procedures complied with the Animal Care and Use Committee of Huazhong Agriculture University (HZAUMO-2016-031). Fertilized embryos were obtained from C57BL/6 females mated with DBA/2. For SCNT, metaphase II (MII) oocytes were collected from 8–10 weeks old female B6D2F1 (C57BL/6×DBA/2) mice. SCNT was performed as previously described [22]. Briefly, the spindle of oocyte was removed by the Piezo-driven (Prime Tech, Japan) enucleation pipette on the Olympus inverted microscope, and the nuclei of cumulus cells were directly injected into the enucleated oocytes. The reconstructed oocytes were cultured in CZB medium for 1 h before activation treatment. The cloned constructs were activated in Ca^2+^-free CZB medium supplemented with 10 mM SrCl_2_ (Sigma, 255521) and 5 μg/ml cytochalasin B (Sigma, C-6762) for 6 h. Cloned and fertilized embryos were cultured in G1-plus medium (Vitrolife, 10132) at 37 °C in an atmosphere of 5% CO_2_ in air.

### Derivation of ntESCs and fertilized-ES cell lines

Derivation of ES cells was performed as previously described with a slight modification [4]. Briefly, expanded blastocysts were transferred into 96 multi-well plate and cultured on feeder layer in ES derivation medium, which contained KO-DMEM (Gibco, 10829018) supplemented with 15% Knockout serum replacement (Gibco, 10828028), MEM Non-Essential Amino Acids Solution (Gibco, 111040050), GlutaMAX™ Supplement (Gibco, 35050061), 2-mercaptoethanol (Specialty media, ES-007-E), EmbryoMax 100X nucleosides (Millipore, ES-008-D), Leukemia inhibitory factor (LIF) (Millipore, ESG1107), 1 μM PD0325901 (Sigma, PZ0162) and 3 μM CHIR99021 (Sigma, SML1046). A week after culture, the outgrowths were passaged.

All other experimental procedures were described in Supplemental Materials.

### Data processing and analysis

Hi-C and ATAC-seq experiments were repeated at least two times and RNA-seq experiments were repeated at least three times. The raw data were processed mainly by using publicly available bioinformatics tools (Table S1). And the written codes for specific analyses are available at https://github.com/v587dexinxin/Nuclear-Transfer-scripts.

The detailed computational procedures were described in Supplemental Materials.

## Results

### Global chromatin accessibility transition during ntESCs reprogramming

**Fig. 1:**
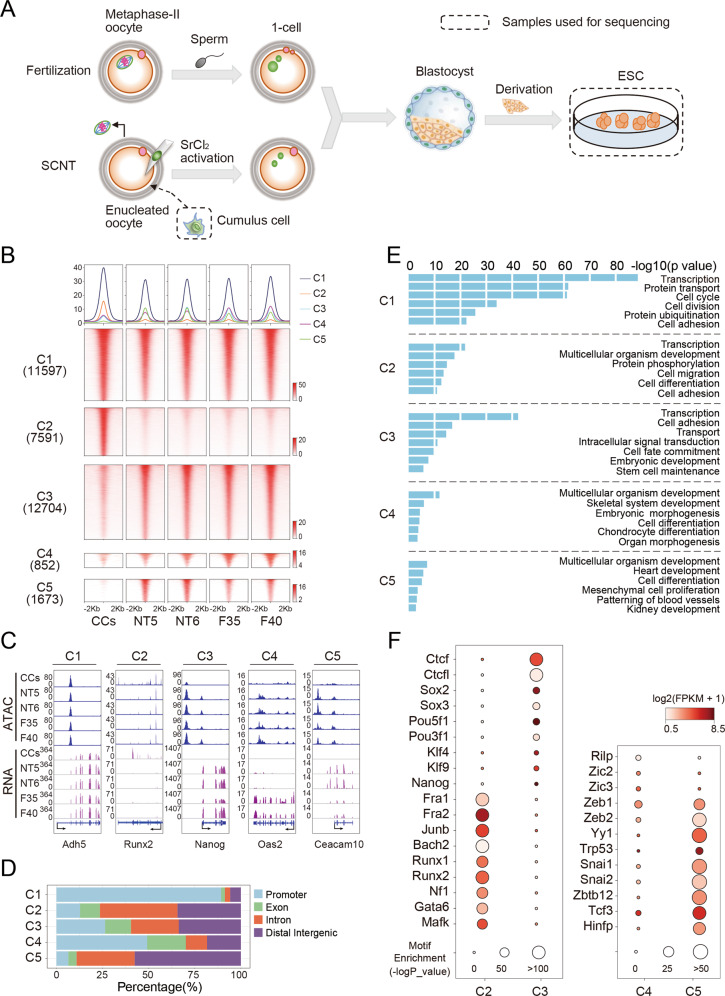
The dynamic changes of global chromatin accessibility of ntESCs. **A** Schematic of fESCs and ntESCs derivation. Blastocysts were obtained from fertilized and SCNT embryos, respectively. fESCs and ntESCs were derived from corresponding blastocysts in vitro and further collected for sequencing. **B** Heatmap of the ATAC-seq profiles. ATAC-seq peaks were classified into three clusters (C1, C2, C3) by K-means according to peak intensities in CCs, NT5, NT6, F35, and F40. The C3 peaks with intensity difference between ntESCs and fESCs were further identified (Supplemental Materials). The peaks with significantly higher intensities in fESCs were defined as C4, and the peaks with reserve intensity trend were defined as C5. Each row represents a locus (ATAC-seq peak center ± 2 Kb), and the red gradient color indicates the ATAC-seq signal intensity. Average tag densities of the identified clusters (midnight blue = C1, coral = C2, sky blue = C3, dark magenta = C4, green= C5) are shown on the top. **C** Examples for ATAC-seq peak clusters. Chromatin regions, *Adh5* (chr3: 138443093–138455499), *Runx2* (chr17: 44,495,987–44,814,797), *Nanog* (chr6: 122,707,489–122,714,633), *Oas2* (chr5: 120,730,333–120,749,853) and *Ceacam10* (chr7: 24,777,206–24,784,657), were selected as examples to show the ATAC-seq and RNA-seq data of CCs, NT5, NT6, F35 and F40 in each clusters. **D** Bar graphs showing the localizations of the ATAC-seq peaks in different clusters. ATAC-seq peaks within TSS ± 1 Kb were considered as promoters and those not located in promoters, exons and introns were labeled as intergenic regulatory elements. **E** Bar plot showing the enriched pathways of ATAC-seq peaks in each cluster (C1, C2, C3, C4 and C5). **F** Dot plots showing the enriched TFs motifs and the gene expressions (*y*-axis) in the different ATAC-seq peak clusters (*x-axis*). Circle size represents the motif enrichment, and the gradient red color indicates the relative gene expressions.

Firstly, we derived ESCs from mouse fertilized and SCNT blastocysts as previously [4] (Fig. 1A). In total, we had established 40 fESC lines and 6 ntESC lines, which were denoted as F1 to F40 and NT1 to NT6, respectively. We selected female fESCs in this study to keep gender consistency. Then, we chose two cell lines with normal karyotypes and morphologies from fESCs and ntESCs, respectively, which are F35, F40, NT5, and NT6 (Fig. S1A). We evaluated their pluripotency by detecting pluripotent and surface marker genes (Fig. S1B, C, Table S2). All four cell lines can produce chimeric mice (Fig. S1D).

The ATAC-seq and RNA-seq datasets show high reproducibility in these four cell lines and the donor cells (cumulus cells, CCs) (Table S3, Fig. S2A, B). The ntESCs show higher correlations with fESCs than those with CCs for chromatin accessibility and gene expression (Fig. S3A), suggesting that chromatin status of reprogrammed ntESCs is more similar with fESCs and departs from CCs. Next, we identified the ATAC-seq peaks [23] (Fig. S3B) and divided them into five clusters: C1 represents the peaks present in all cell types, C2 represents the CC-specific peaks, C3 represents the ESC-specific peaks, C4 represents the ESC-specific peaks with fESCs signals significantly higher than ntESCs signals, and C5 represents the ESC-specific peaks with ntESCs signals significantly higher than fESCs signals (Fig. 1B, C). Compared to C1 and C4, most C2, C3 and C5 peaks are located outside the promoter regions (Fig. 1D), indicating that the distal regulatory elements are remodeled upon SCNT. The genes in C1 are related to universal biological processes, the genes in C2 are mainly related to multicellular organism development and cell differentiation, the genes in C3 are mainly related to embryonic development and stem cell maintenance, the genes in C4 are related to embryonic morphogenesis and cell differentiation, and the genes in C5 are related to mesenchymal cell proliferation, development of heart and kidney (Fig. 1E). The TF motif enrichment analysis shows that the somatic TF motifs (*Junb, Runx1/2* and *Fra1/2*) are enriched in C2 while the pluripotent TF motifs (*Pou5f1, Klf4* and *Nanog*) are relatively enriched in C3 (Fig. 1F, Fig. S3C). Moreover, many lineage-specific TFs are detected in C5, such as *Snai1/2*, which are involved in the mesoderm commitment of ESC differentiation [24–26] (Fig. 1F). Collectively, ntESCs and fESCs show high similarities in chromatin accessibility with subtle but significant differences, which may be involved in the tissue and organ development during ntESC differentiation.

### Global 3D genome reprogramming of ntESCs

**Fig. 2:**
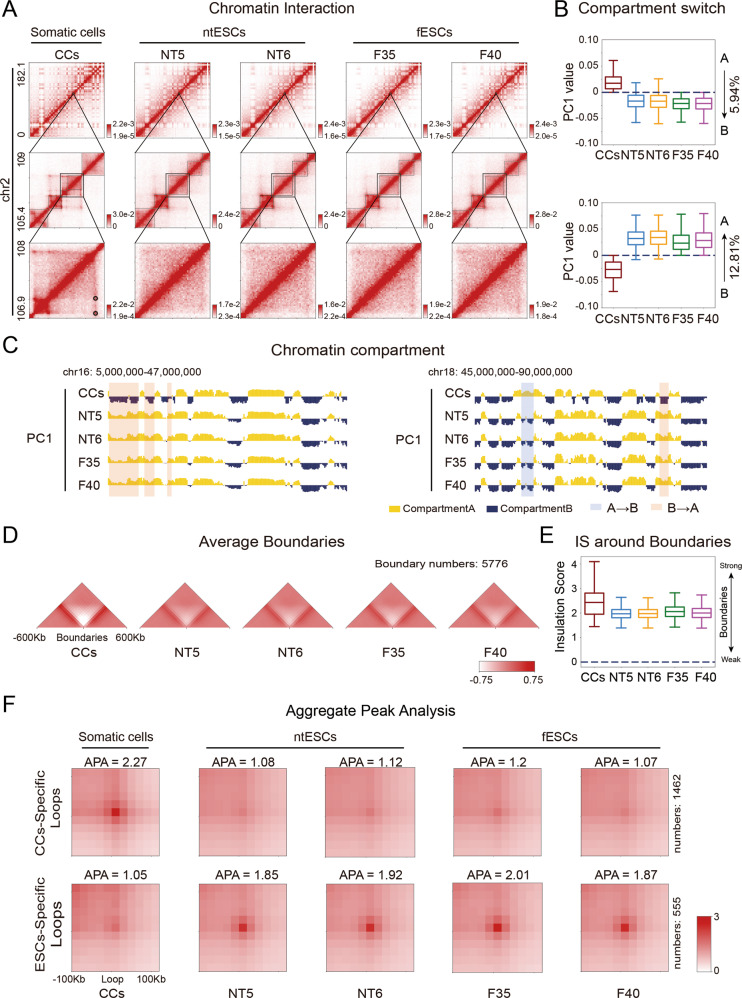
Global 3D genome organization of ntESCs. **A** Hi-C heatmaps showing the differences and similarities among CCs, NT5, NT6, F35, and F40 at different levels. The heatmaps were shown in 200 Kb, 40 Kb. and 20 Kb resolutions respectively from top to bottom subfigures. **B** Boxplots showing the compartmental transitions, A-to-B (on the top) and B-to-A (on the bottom), from CCs to fESCs. The percentage of each transition type is shown on the right of the graph. **C** Chromatin compartments represented by the first principal component (PC1) values derived from PCA analyses in CCs, NT5, NT6, F35 and F40. Positive and negative PC1 values represent compartment A (yellow) and compartment B (dark-blue) regions, respectively. The compartmental transitions (A-to-B and B-to-A) between CCs and the other four cell lines are shown in the light-blue and light-red colors, respectively. **D** Heatmaps of average interaction frequencies for all boundaries as well as their nearby regions (± 600 Kb) at CCs, NT5, NT6, F35 and F40 (40 Kb resolution). **E** Boxplot showing the insulation scores at the boundaries in each cell type. **F** APA (Aggregate Peak Analysis) plots for chromatin loops. The number of loops is displayed on the right.

We next performed Hi-C experiments to investigate the 3D genome reprogramming. The high correlations between replicates indicate the high reproducibility in five cell types (Fig. S2C, D). Compared to previously published Hi-C data derived from SCNT blastocysts [18], our Hi-C data show higher quality (Fig. S4A), which helps us to further study 3D chromatin structures and their biological functions. Firstly, compared to ntESCs and fESCs, the CCs show stronger proximal interactions (~80 Kb) but weaker distal interactions (~0.4 Mb) overall (Fig. S4B). We then analyzed the 3D genome organization in three layers: compartment, TAD and loop. The ntESCs show high similarities to fESCs but considerable difference to CCs (Fig. 2A). Correspondingly, both the PC1 correlation coefficients and the interaction correlation heatmaps show that ntESCs are globally different from CCs and highly resemble fESCs at the compartment level (Fig. S4C, D). Further analyses show that ntESCs and fESCs have weaker segregated compartments A and B than CCs (Fig. S4E). There is a subtle but no significant difference in the compartmentalization strength between ntESCs and fESCs (Fig. S4F). Based on these results, we focused on the changes in ntESCs compared to CCs and fESCs. About 12.81% of ntESCs chromatin regions undergo switches from CC-specific compartment B to ESC-specific compartment A, and 5.94% undergo switches from the CC-specific compartment A to ESC-specific compartment B (Fig. 2B, C). These results suggest that massive ntESCs compartments are reorganized into ESC-like structures during reprogramming.

We investigated the dynamics of TADs and loops. Though the TAD boundaries show some stability in all cell types, the TADs in CCs generally become weaker and exhibit much higher insulation effects than those in ntESCs and fESCs, indicating the overall weakening of boundary insulation during reprogramming (Figs. 2A, D, E, Fig. S4G, H). Consistently, 1462 CC-specific loops disappear in ntESCs and fESCs (Fig. 2A, F), while 555 fESC-like loops are built in ntESCs. Overall, the ntESCs show higher similarities to fESCs than CCs at both TAD and loop levels, indicating the global 3D genome reprogramming of ntESCs.

### Rewiring of 3D genome in pluripotent and somatic transcription factors

**Fig. 3:**
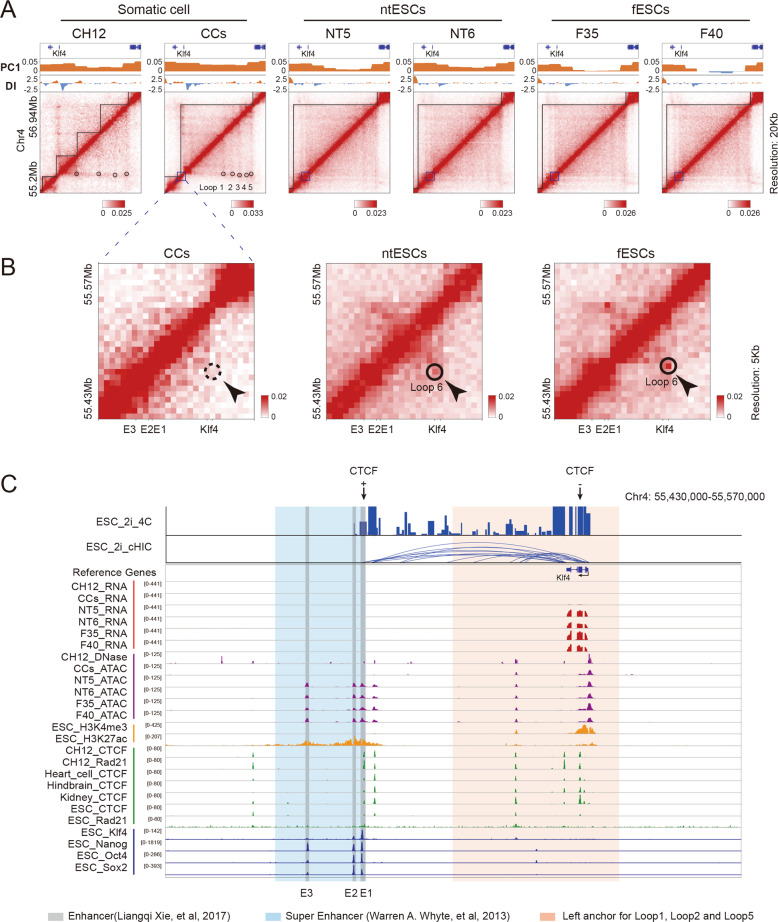
Rewiring of chromatin loops around the *Klf4* gene. **A** Heatmaps showing the normalized Hi-C interaction frequencies and corresponding PC1 and directionality index (DI) values around the *Klf4* gene (Chr4: 55,200,000-56,940,000) at 20 Kb resolution for CH12 [27], CCs, NT5, NT6, F35 and F40. **B** Heatmaps showing the normalized Hi-C interaction frequencies around the *Klf*4 gene and its enhancer regions [31] at 5 Kb resolution for CCs, ntESCs and fESCs. **C** The illustration of gene expression, epigenomic signals, transcription factor binding signals and chromatin interactions around the *Klf4* gene. The region is the same as shown in (**B**). The regions in light red are the left anchors of loop 1/2/5 in CCs and the regions in light blue and gray are the reported super enhancer [30] and the enhancers [31].

We investigated the dynamic changes of chromatin structures in the regions coding for pluripotent TFs. Several chromatin loops (denoted as loop 1/2/3/4/5 in CCs) anchored around gene *Klf4* disappear in ntESCs and fESCs compared to somatic cells (CCs and CH12 [27]) (Fig. 3A). Then, we called the loops at higher 5Kb resolution and detected a short-distance chromatin loop (denoted as loop 6) appearing around gene *Klf4* in ntESCs and fESCs (Fig. 3B). To further investigate the potential functions of this chromatin-loop rewiring, we combined our datasets with publicly available datasets, in which 4C-seq [28], cHi-C [29] and ChIP-seq of various proteins in ESCs were used as the references for ntESCs and fESCs, and the ChIP-seq (CTCF and Rad21) and DNase-seq in various types of somatic cells were used as references for CCs (Table S4). Interactions between gene *Klf4* and enhancers (E1 and E2) are detected by both 4 C and cHi-C in ESCs, which are highly co-localized with loop 6 in ntESCs and fESCs (Fig. 3C). E1 and E2 are enriched with pluripotent TF binding sites and H3K27ac domains, consistent with that E1, E2 and E3 together are composed of super enhancer in ESCs [30, 31]. These results imply that loop 6 in ntESCs and fESCs probably regulates pluripotency by connecting super enhancer and gene *Klf4*. By contrast, the left anchor of loops 1, 2 and 5 in CCs localizes between gene *Klf4* and the region of super enhancer, implying that these loops may separate gene *Klf4* from nearby enhancers.

Moreover, the *Sox2* region switches from compartment B-to-A during reprogramming, and the boundary insulation and chromatin loops also undergo significant changes (Fig. S5). The *Nanog* region in ntESCs and fESCs exhibits slightly stronger compartment-A signals than those in CCs and *Oct4* region exhibits slight change in local chromatin structure (Fig. S5). In addition, the region of somatic-specific TF *Runx1* undergoes significant changes, with disappearance of several chromatin loops and remodeling of TAD boundaries (Fig. S5). Collectively, chromatin regions coding pluripotent and somatic TFs exhibit rewiring of 3D genome during ntESC reprogramming.

### Abnormal chromatin structure organization of ntESCs

**Fig. 4:**
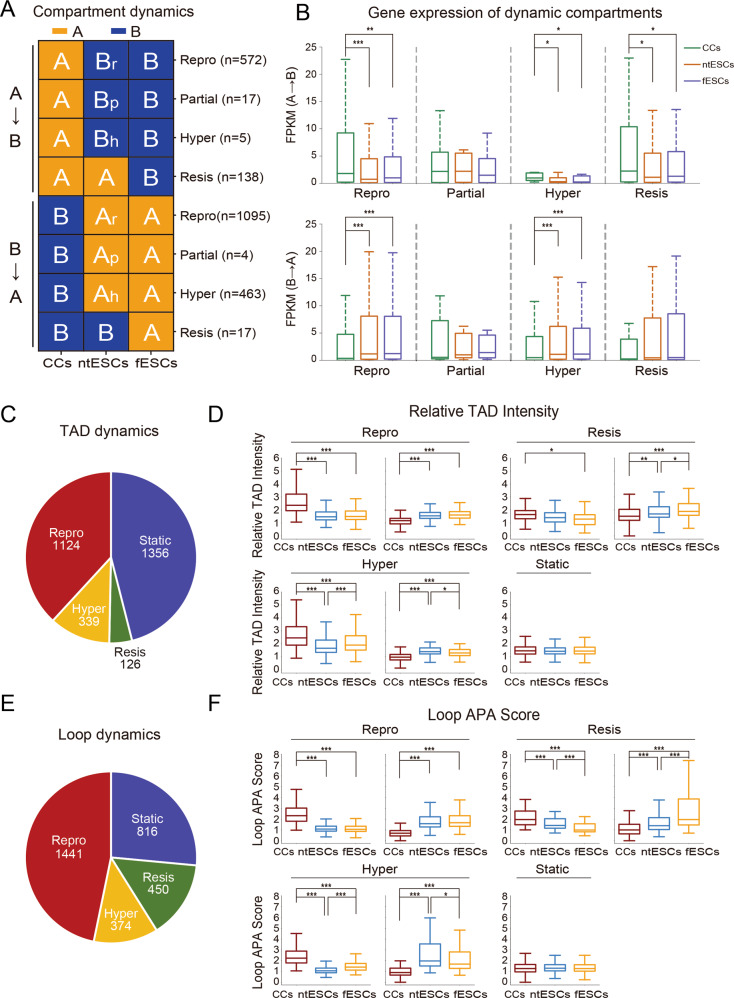
Abnormal chromatin structures in ntESCs. **A** The classifications of compartment dynamics. The number of compartments in each group is listed. The ‘Repro’ (Reprogrammed), ‘Partial’, ‘Hyper’ and ‘Resis’ (Resistant) were defined based on the compartmental states among CCs, ntESCs and fESCs. **B** Boxplots showing the average gene expressions in CCs, ntESCs, and fESCs for each group of compartmental states defined in Fig. 4A. Statistical analysis was performed by using the Wilcoxon rank-sum statistic test. *** *P* < = 0.0005, ** 0.0005 < *P*-value < = 0.005, and * 0.005 < *P* < = 0.05. **C**, **E** Pie charts showing the classifications of ‘static’, ‘Repro’ (Reprogrammed), ‘Resis’ (Resistant) and ‘Hyper’ TAD and Loop dynamics, respectively. Each group of TAD and Loop dynamics was defined based on the relative TAD intensity (RTI) [57] and Loop APA score [27], respectively, among CCs, ntESCs and fESCs. The number of TAD and Loop dynamics in each group is listed. **D**, **F** Boxplots showing the values of the relative TAD intensity (RTI) and Loop APA score for each group of TAD and Loop dynamics, respectively. The Statistical analysis is consistent with the method in (**B**).

We observed that a few chromatin regions in ntESCs do not switch to the compartmental states in fESCs (Fig. S6A). Then we grouped these abnormal chromatin regions into four classes: (1) ‘Repro’ (Reprogrammed), indicating that these compartments in ntESCs switch completely to the states in fESCs; (2) ‘Partial’, indicating that the switched compartments in ntESCs show the intermediate PC1 values between CCs and fESCs; (3) ‘Hyper’, indicating that the switched compartments in ntESCs show higher PC1 values than those in fESCs; (4) ‘Resis’ (Resistant), indicating that the compartments in ntESCs largely show the same states of CCs (Fig. 4A, Fig. S6A). There are no significant differences in overall gene expressions between ntESCs and fESCs in each group (Fig. 4B). Especially, in resistant A-to-B compartments, the gene expression levels in ntESCs and fESCs are significantly lower than those in CCs (Fig. 4B), which may be due to the fact that gene expression and compartment change are not always consistent [32].

We then investigated the TAD/loop dynamics by classifying the switches into four groups: ‘Repro’, ‘Resis’, ‘Hyper’ and ‘Static’. The first three groups are defined similarly to those in compartment dynamics, and the group ‘Static’ represents TADs/loops without significant changes among CCs, ntESCs and fESCs. (Fig. 4C–F and Fig. S7A, B). Collectively, we defined the ‘Partial’, ‘Hyper’ and ‘Resis’ compartments as well as ‘Resis’ and ‘Hyper’ TADs/loops as being abnormal regions. In general, the GO analyses show that the genes located in abnormal structures are mainly involved in apoptotic signaling pathways, the development of tissues and organs, embryonic morphogenesis and others (Fig. S6B). Taken together, these results indicate that aberrant structures in these layers have the potential to affect the biological and differentiation processes of ntESCs.

### Abnormal chromatin structures impair the differentiation process of ntESCs

**Fig. 5:**
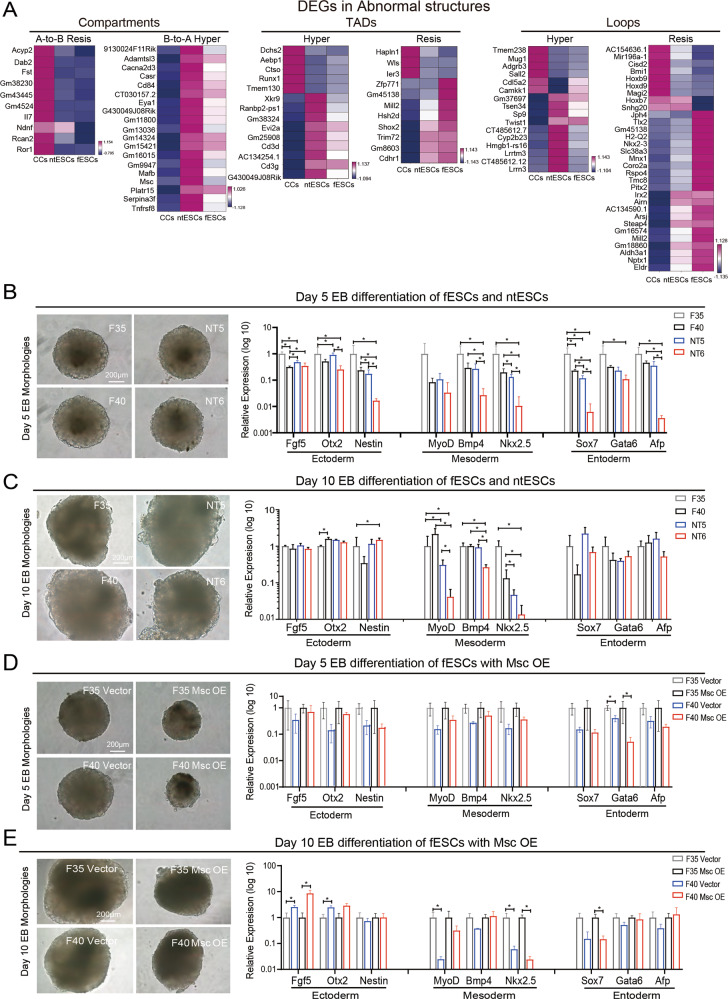
Abnormal chromatin structures impair the differentiation process of ntESCs. **A** Heatmaps showing the examples of the differential expression genes (adjusted p-value < 0.05) between ntESCs and fESCs related to the abnormal chromatin structures at three layers: Compartment, TAD and Loop. The gene expressions (FPKM) in each row were normalized by using Z-score. **B** The Day 5 EB morphologies of ntESCs and fESCs (left), scale bar, 200 µm; qRT-PCR analysis of three-layer genes (right) in day 5 EBs, mean ± SD (*n* = 3). **C** The Day 10 EB morphologies of ntESCs and fESCs (left), scale bar, 200 µm; qRT-PCR analysis of three-layer genes (right) in day 10 EBs, mean ± SD (*n* = 3). **D** The Day 5 EB morphologies of fESCs with empty *vector* and *Msc* OE (left), scale bar, 200 µm; qRT-PCR analysis of three-layer genes (right) in day 5 EBs, mean ± SD (*n* = 3). **E** The Day 10 EB morphologies of fESCs with empty *vector* and *Msc* OE (left), scale bar, 200 µm; qRT-PCR analysis of three-layer genes (right) in day 10 EBs, mean ± SD (*n* = 3). The Wilcoxon rank-sum test was used in *P*-value calculations. * 0.01 < = *P* < 0.05, ** 0.001 < = *P* < 0.01 and *** *P* < 0.001 in (**B**–**E**).

It was reported that some cloned mice suffered from diseases in their development of immune system and organs [10, 11]. Intriguingly, many differentially expressed genes (DEGs) between ntESCs and fESCs are detected in the abnormal structures, and many of these DEGs are vital for the development of tissue and organs, such as *Dab2*, *Rcan2, Msc and* others [33–36] (Fig. 5A). Subsequently, to confirm whether there are abnormalities in the differentiation process of ntESCs, we performed EB induced differentiation on ntESCs and fESCs. Although there is no difference in the morphologies of Day 5 and Day 10 EBs between ntESCs and fESCs, the genes of three germ layers show some abnormal expressions in ntESCs, especially in NT6 (Fig. 5B, C). Spontaneously contracting cells can be detected in EB after 7–12 days of differentiation, which are cardiomyocytes developed from mesoderm [37]. And there are 73% (11/15) spontaneously beatings in day 10 EBs detected in F35, 67% (12/18) in F40, 47% (7/15) in NT5 and 20% (3/15) in NT6 (Supplement video 1–4). The lower beating percentages in ntESCs suggest that there are abnormalities in tissue and/or organ development.

To verify whether the genes in abnormal structures can impair stem cell differentiation, we select the gene *Msc* since it is located in ‘Hyper’ compartment A with higher expression in ntESCs (Fig. 5A and S6A). *Msc* is reported to be involved in the development of various muscles [36, 38–42]. Considering the complexity of the rescue experiment in ntESCs due to numerous DEGs, we overexpressed the gene *Msc* in fESCs. Compared to empty *vector* cell lines, the fESCs over-expressing *Msc* (*Msc* OE) show normal ESC morphologies and pluripotent gene expressions (Fig. S8A), but the *Msc* OE cell lines on day 5 have smaller EBs and slightly lower gene expressions of three germ layers (Fig. 5D). There are none spontaneously beating in day 10 EBs in *Msc* OE cell lines (F35 *Msc* OE: 0/24; F40 *Msc* OE: 0/24) and more than 80% spontaneously beating in day 10 EBs in *vector* cell lines (F35 *vector*: 20/24; F40 *vector*: 22/24) (Supplement video 5–8). Meanwhile, the expressions of mesoderm genes are lower in *Msc* OE cell lines in day 10 EBs (Fig. 5E). These results suggest that the gene *Msc*, located in the abnormal chromatin regions in ntESCs, has no effect on the self-renewal and pluripotency of ESCs, but impairs the differentiation process of ESCs.

### H3K9me3 modification may be involved in abnormal chromatin structures of ntESCs

**Fig. 6:**
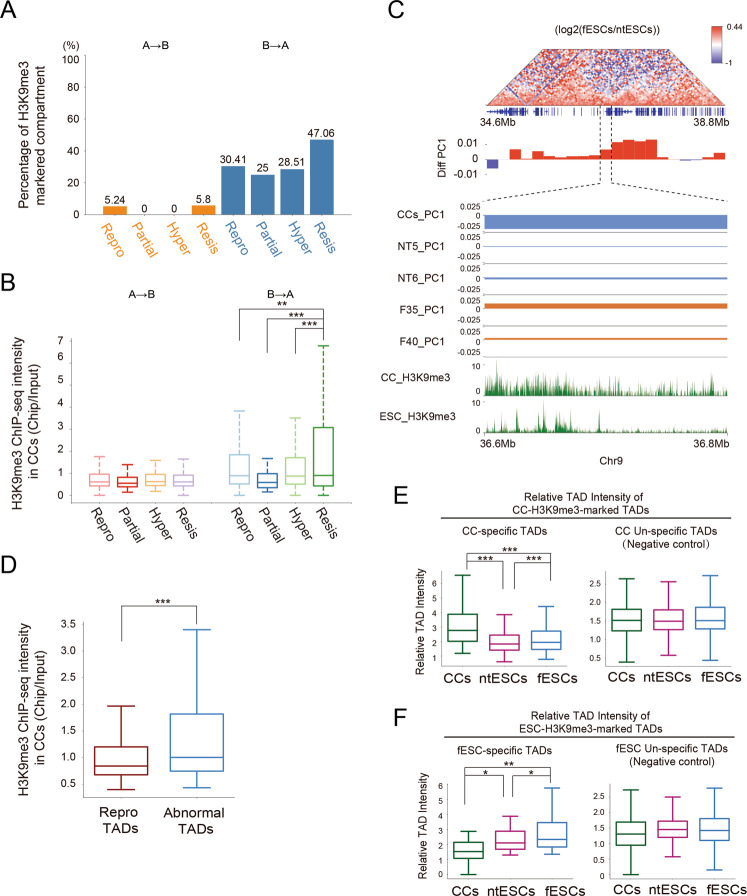
H3K9me3 in Abnormal chromatin structures in ntESCs. **A** The bar chart showing the proportion of the H3K9me3-marked compartments in each group in Fig. 4A. **B** Boxplot showing the average H3K9me3 Chip-seq intensity (Chip/Input) in CCs for each group of compartment dynamics (200 Kb resolution). **C** The differential interaction frequencies (log2(fESCs/ntESCs)) and corresponding H3K9me3 signals around a B-to-A resistant compartment region. The bottom tracks show the H3K9me3 signals in CCs [18] and ESC [58] respectively within a B-to-A resistant region (Chr9: 36.6 Mb – 36.8 Mb). **D** Boxplot showing the average Chip-seq intensity (Chip/Input) of H3K9me3 in CCs for the ‘Repro’ and ‘Abnormal’ TADs (including ‘Resis’ and ‘Hyper’). **E** Boxplot showing the average relative TAD intensity (RTI) of the H3K9me3-marked CC-specific TADs. The CC-unspecific TADs are also shown as control. **F** Boxplot showing the average relative TAD intensity (RTI) of the H3K9me3-marked ESC-specific TADs. The ESC-unspecific TADs are also shown as control. Statistical analysis was performed by using the Wilcoxon rank-sum statistic test. *** *P* < = 0.0005, ** 0.0005 < *P* value < = 0.005, and * 0.005 < *P* < = 0.05.

Since it is reported that somatic-specific H3K9me3 modifications hindered somatic cell reprogramming [43–46], we wondered whether the H3K9me3 modifications were associated with the abnormal structures in ntESCs. Hence, we followed a previous study to calculate H3K9me3-marked regions [18]. Our results show that less than 5.8% regions of A-to-B abnormally switched compartments are marked by H3K9me3 in CCs whereas 25–47.06% regions of each group of B-to-A abnormally switched compartments are marked by H3K9me3 in CCs (Fig. 6A, Fig. S9). Actually, the B-to-A resistant switch compartments show significantly stronger H3K9me3 signals than other cases (Fig. 6B, C). Similarly, the H3K9me3 signals in abnormal TADs are significantly higher than those in reprogrammed TADs in CCs (Fig. 6D). These results together imply that H3K9me3-marked regions may be involved in impeding the rearrangement of chromatin structures during reprogramming.

To explore the question whether the abnormal TADs detected in SCNT 2-cell embryos still show abnormal structures in ntESCs, we then identified the H3K9me3-marked TADs in CCs as previously [18]. Interestingly, the H3K9me3-marked CC-specific TADs undergo dramatic rearrangements during reprogramming, with even lower intensities in ntESCs than those in fESCs (Fig. 6E). This result implies that these H3K9me3-marked CC-specific TADs, which are initially difficult to disassemble in SCNT 2-cell embryos [18], can be remodeled but are hard to be remodeled to the exactly same fESCs states in ntESCs. Since the embryo-specific H3K9me3 modifications play vital roles in early embryogenesis and cell fate determination [47]. We next identified the H3K9me3-marked TADs in ESCs. The results show that the H3K9me3-marked fESC-specific TADs show significantly lower intensities in ntESCs (Fig. 6F), suggesting that these fESC-specific TADs are also hard to be remodeled to the exactly same fESCs states in ntESCs. Taken together, the results imply that H3K9me3 not only impedes the disassemble of somatic chromatin structures in SCNT 2-cell stages but also may affect the exact establishment of chromatin structures in ntESCs.

### Comparison of 3D genome between ntESCs and iPSCs

**Fig. 7:**
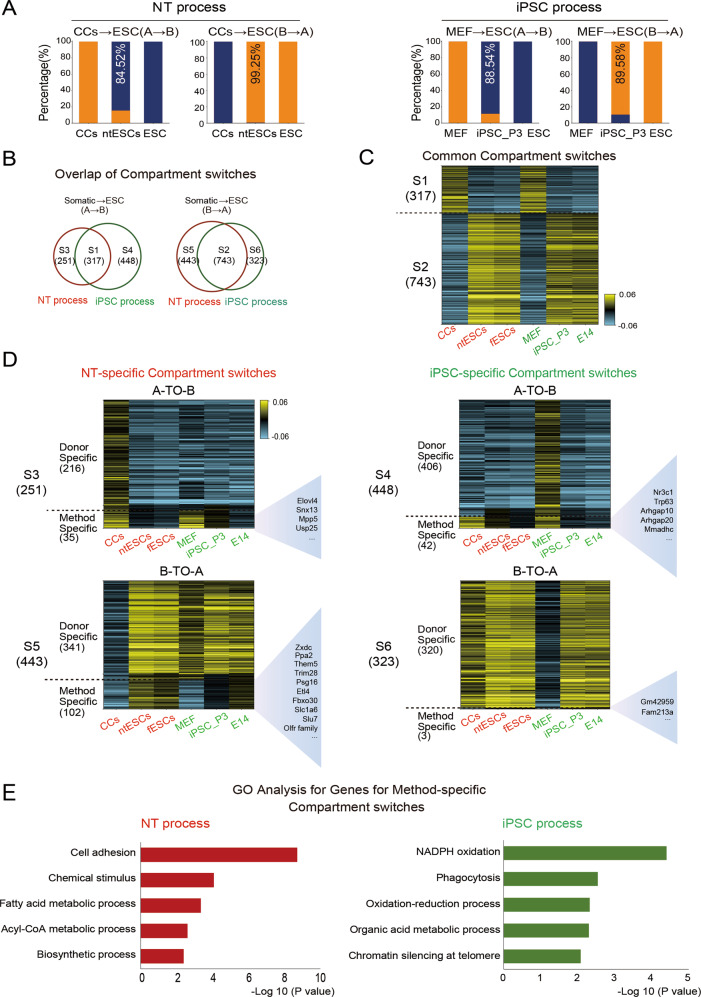
Comparison of 3D chromatin structures between ntESCs and iPSCs. **A**, Barplots showing the percentages for those A-to-B and B-to-A compartment switches from CCs to ESC in NT process and from MEF to ESC in iPSC process (200 Kb resolution). The percentage numbers are shown in ntESCs and iPSC. ESC represents the same chromatin compartmentalization between fESCs and E14. **B** The Venn diagrams show the overlap of compartment switches between NT process and iPSC process. The percentages and classification labels are also shown. **C** The heatmap showing the PC1 values of the common compartment switches between two processes. The numbers of A-to-B and B-to-A reprogrammed compartments in the NT and iPSC processes are shown in the left side of heatmap respectively. **D** The heatmap shows the PC1 values of NT-specific (left) and iPSC-specific (right) compartment switches. The numbers of NT-specific A-to-B (left, top), NT-specific B-to-A (left, bottom), iPSC-specific A-to-B (right, top) and iPSC-specific B-to-A (right, bottom) reprogrammed compartments are shown on the left side of the heatmaps, respectively. ‘Donor specific’ means that there are different compartmental states between CCs and MEF, but the NT process or iPSC process is reprogrammed. ‘Method specific’ means that there are same compartmental states between CCs and MEF, but different compartmental states between ntESCs and iPSC. The differentially expressed genes between ntESCs and iPSC related to method-specific compartment switches were displayed on the right side of each heatmap. **E** The GO analyses on the genes related to Method-specific categories in the NT-specific and iPSC-specific compartment switches.

To compare the reprogramming processes between ntESCs and iPSCs at the 3D genome level, we integrated the public datasets for comparisons [21]. The results show that both p3 and p20 iPSCs are highly similar to ntESCs and fESCs (Fig. S10A, B). Since p3 iPSCs show slightly higher similarities to ntESCs in compartments, we selected the p3 iPSCs for the following comparisons. Based on the same chromatin compartmentalization between fESCs and E14 (Fig. S10C), 84.52% of CC-specific compartments A switch to ESC-specific compartments B and 99.25% of CC-specific compartments B switch to ESC-specific compartments A in the NT process, while the iPSC process shows 88.54% A-to-B switches and 89.58% B-to-A switches (Fig. 7A). And the two processes show consistency between gene expression and compartment dynamics (Fig. S10D).

To further investigate the differences in compartment switch between NT and iPSC processes, we separated compartments into common switch (S1 and S2) and different switches in the two processes (S3–S6) (Fig. 7B, C). Moreover, we also observed differently reprogrammed compartments can be further classified into donor-specific and method-specific switches (Fig. 7D). Actually, the donor-specific compartment switches dominate the numbers of switching differences between the two processes, with consistent gene-expression trends (Fig. S10E). These results indicate that most chromatin regions can be successfully reprogrammed to ESC-like states even though they have different compartment states in donor cells. However, a few method-specific compartments share the same states in donor cells, but they exhibit different states between ntESCs and iPSCs. Especially, we detected that the gene *Trim28*, which is thought to be an transition-resisting factor in the iPSCs induction [48, 49], located in method-specific compartment B-to-A switch of the NT process (Fig. 7D). Besides, the GO analysis on the genes in method-specific switches show that the iPSCs process is mainly associated with NADPH oxidation and oxidation-reduction process, and the NT process is associated with cell adhesion and chemical stimulus (Fig. 7E). These results are consistent with previous reports that NT and iPSC have different biological and metabolic processes, with the iPSCs process needing the switch from oxidative phosphorylation to glycolysis [50, 51]. Moreover, a small number of resistant compartments are observed in NT and iPSC processes, which are mainly not overlapped (Fig. S10F, G). Especially, compared to the NT process, the iPSCs process harbors more resistant regions in B-to-A compartment switches. Taken together, NT and iPSCs processes show similar 3D genome reprogramming overall, and even the donor-specific compartments can be reprogrammed to the ESC-like states. The minor differences between NT and iPSCs processes can also be observed through these chromatin regions share the same compartment states in different donor cells.

## Discussion and conclusion

The generation of pluripotent stem cells from cloned blastocysts, especially the successful derivation of human ntESCs [51], holds great promise for regenerative medicine. Though many methods are used to improve the development of cloned early embryos, most of them still cannot develop into full term [51]. And even after birth, the cloned animals always carry some diseases [10, 12, 13]. These results indicate that there are still many defects in the SCNT blastocysts that hinder the development of post-implantation embryos. It is known that chromatin 3D structure is an important layer of gene regulation. Two works already revealed that variations in 3D structures impact the development of SCNT embryos by impairing the expression of key genes [18, 19]. However, these works mainly focused on the SCNT embryos developmental stage, leaving the chromatin structure reprogramming of their developmental potential largely unknown. In this work, we aimed at investigating the abnormities by comparing ntESCs to fESCs in the regulatory layers of chromatin accessibility and chromatin 3D structure.

**Fig. 8:**
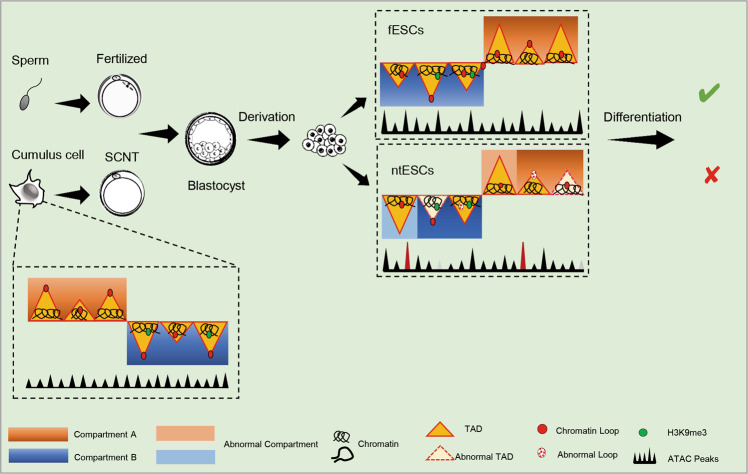
A schematic model showing the 3D genome reorganization during SCNT reprogramming. ntESCs are used to replace SCNT blastocysts to build high-quality Hi-C data. Significant differences in the chromatin accessibility and chromatin structures at all three layers (compartments, TAD boundaries and loops) in ntESCs. And abnormal chromatin structures impair the differentiation process of ntESCs. H3K9me3-marked TADs show lower intensities and may affect the exact establishment of chromatin structures in ntESCs.

We show that the global chromatin accessibility of somatic cells is successfully remodeled to ESC-like state in ntESCs. Consistent with previous studies of chromatin accessibility in SCNT embryos and iPSCs generation [30, 33], our results show that the distal regulatory regions of ntESCs generally undergo dynamic reprogramming. In addition, many somatic-specific ATAC-seq peaks are reported to resist reprogramming in SCNT early embryos, such as AP-1 and *Elf1* [20, 52], but our results show that these somatic-specific peaks are reprogrammed successfully in ntESCs. Nevertheless, we also detect that there are a few differential peaks between ntESCs and fESCs (Fig. 8). Especially, the ntESC-specific peaks are highly enriched with the motifs of *Snai1/2* and *Tcf3*, which are reported to be involved in mesodermal commitment during ESC differentiation [24–26]. The above results imply that the obstacles of SCNT embryo stages have been overcome while new abnormalities in chromatin accessibility emerged in ntESCs.

The 3D structures of somatic cells undergo remodeling to ESC-like structures at compartment, TAD and loop levels, but still have some abnormal chromatin structures in ntESCs (Fig. 8). In some human diseases, there is a link between chromatin 3D structures and pathogenic mechanisms, with variations in chromatin 3D structures causing the expression alterations of related genes [53, 54]. Consistently, we detected many DEGs in the abnormal regions of ntESCs and demonstrated that the ectopic expression of key genes could impair the ntESCs differentiation process (Fig. 8). The H3K9me3-marked TAD structures in somatic cells have been proved to be dissolved in SCNT 2-cell embryos hardly [18, 43, 46]. Our study revealed that H3K9me3 in ntESCs was involved in incorrect compartment switches and incomplete TAD rebuilding (Fig. 8). The above results indicate that there is a unique process at each reprogramming stage. Overexpression of *Kdm4d* (H3K9me3 demethylase) can partially reduce the TAD intensity in SCNT embryos and rescue their development [18], however, whether overexpression of *Kdm4d* can rescue the abnormal structures as well as development potentials in ntESCs need to be further studied. Overall, our study provides a complementary perspective to the relationship between H3K9me3 and chromatin reconstruction in the SCNT reprogramming.

Finally, our results show that ntESCs and iPSCs have highly similar 3D structures to each other as well as general ESCs. It is reported that somatic cell reprogramming efficiency depends on somatic cell types and methods [21, 55, 56]. And SCNT and iPSCs display completely different biological and metabolic processes [50]. Consistently, our results also show some differences in 3D genome reprogramming between ntESCs and iPSCs, with most of them being caused by somatic cell types and a small part of them being caused by different reprogramming mechanisms. Limited by public iPSCs datasets, it is hard for us to thoroughly explore whether there are key factors involved in regulating the mechanism-related differential reprogramming of 3D structures. Further rigorous comparisons between ntESCs and iPSCs in 3D genomes are exciting topics in the future. There is no doubt that utilizing 3D genome to explore respective features can not only help us obtain a better understanding of reprogramming but also promote the development of therapeutic applications.

## Supplementary information

Reproducibility checklist

Supplemental Materials

Table S1

Table S2

Table S3

Table S4

Supplement video1 F35 Day10 EB

Supplement video2 F40 Day10 EB

Supplement video3 NT5 Day10 EB

Supplement video4 NT6 Day10 EB

Supplement video5 F35 Msc OE Day10 EB

Supplement video6 F40 Msc OE Day10 EB

Supplement video7 F35 Vetor Day10 EB

Supplement video8 F40 Vetor Day10 EB

## Data Availability

All data have been deposited to the Genome Sequence Archive in BGI Data Center (https://bigd.big.ac.cn/gsa/) with the GSA accession number CRA001552.
