# Correction: Defective chromatin architectures in embryonic stem cells derived from somatic cell nuclear transfer impair their differentiation potentials

**DOI:** 10.1038/s41419-021-04426-9

**Published:** 2021-12-06

**Authors:** Dan-Ya Wu, Xinxin Li, Qiao-Ran Sun, Cheng-Li Dou, Tian Xu, Hainan He, Han Luo, Haitao Fu, Guo-Wei Bu, Bingbing Luo, Xia Zhang, Bin-Guang Ma, Cheng Peng, Yi-Liang Miao

**Affiliations:** 1 Institute of Stem Cell and Regenerative Biology, College of Animal Science and Veterinary Medicine, Huazhong Agricultural University, 430070 Wuhan, Hubei China; 2 Key Laboratory of Agricultural Animal Genetics, Breeding and Reproduction (Huazhong Agricultural University), Ministry of Education, 430070 Wuhan, Hubei China; 3 Hubei Key Laboratory of Agricultural Bioinformatics, College of Informatics, Huazhong Agricultural University, 430070 Wuhan, Hubei China; 4 Center for Life Sciences, School of Life Sciences, Yunnan University, 650500 Kunming, China; 5 Hubei Hongshan Laboratory, 430070 Wuhan, China

**Keywords:** Embryonic stem cells, Reprogramming, Stem-cell differentiation

Correction to: *Cell Death and Disease* 10.1038/s41419-021-04384-2, published online 16 November 2021

The original version of this article unfortunately contained a mistake in an author affiliation. The correct affiliation of Xinxin Li is Hubei Key Laboratory of Agricultural Bioinformatics, College of Informatics, Huazhong Agricultural University, 430070 Wuhan, Hubei, China. The original article has been corrected.

