# Supplementary material for: Defective chromatin architectures in embryonic stem cells derived from somatic cell nuclear transfer impair their differentiation potentials
Source: Cell Death Dis. 2021 Nov 16;12(12):1085. doi: 10.1038/s41419-021-04384-2 (PMC8595669; doi:10.1038/s41419-021-04384-2)
Supplement: Supplementary file 2 — Supplemental Materials [file 41419_2021_4384_MOESM2_ESM.pdf]

Supplemental Materials

Defective chromatin architectures in embryonic stem cells derived from somatic cell nuclear transfer impair their differentiation potentials

Dan-Ya Wu, Xinxin Li, Qiao-Ran Sun, Cheng-Li Dou, Tian Xu, Hainan He, Han Luo, Haitao Fu, Guo-Wei Bu, Bingbing Luo, Xia Zhang, Bin-Guang Ma, Cheng Peng and Yi-Liang Miao

Supplemental Figures

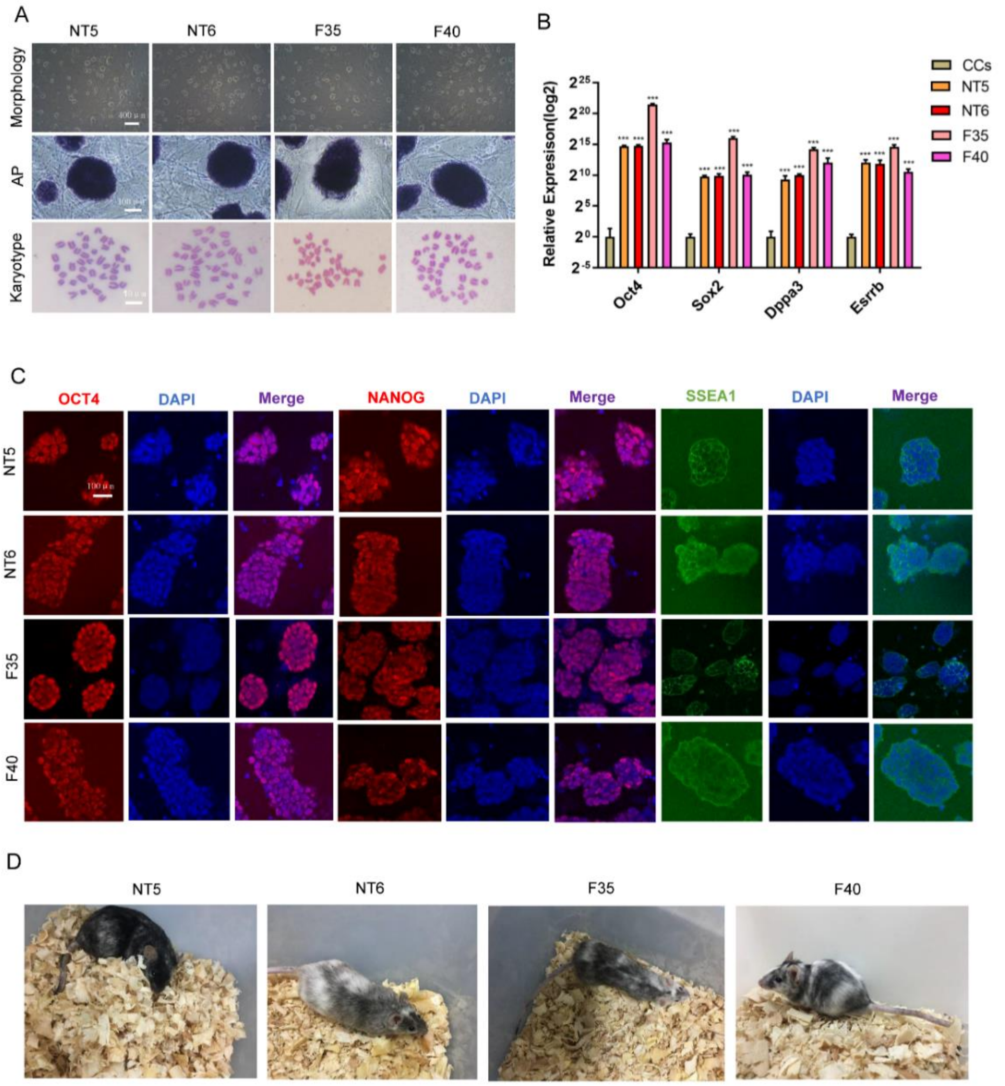

Figure S1 Characteristics of ESCs derived from fertilized and SCNT embryos

A, Morphology (top), alkaline phosphatase (middle) and karyotype (bottom) of NT5, NT6, F35 and F40. Scale bars are 400 μm, 100 μm and 10 μm, respectively. **B**, Quantitative PCR analyses of the pluripotent genes Oct4, Sox2, Dapp3 and Esrrb in NT5, NT6, F35 and F40. Relative mRNA expression was normalized to GAPDH and cumulus cells (CCs) were used as negative control. The experiments were performed in triplicate (mean±SD; n=3). Statistical analysis was performed by using T-test. * $0.01 <= P < 0.05$, ** $0.001 <= P < 0.01$ and *** $P < 0.001$. **C,** Immunofluorescence staining of pluripotent markers Oct4, Nanog and SSEA-1 in NT5, NT6, F35 and F40. Nuclei were stained with DAPI. Scale bar is 100 μm. **D,** Chimera mice derived from NT5, NT6, F35 and F40.

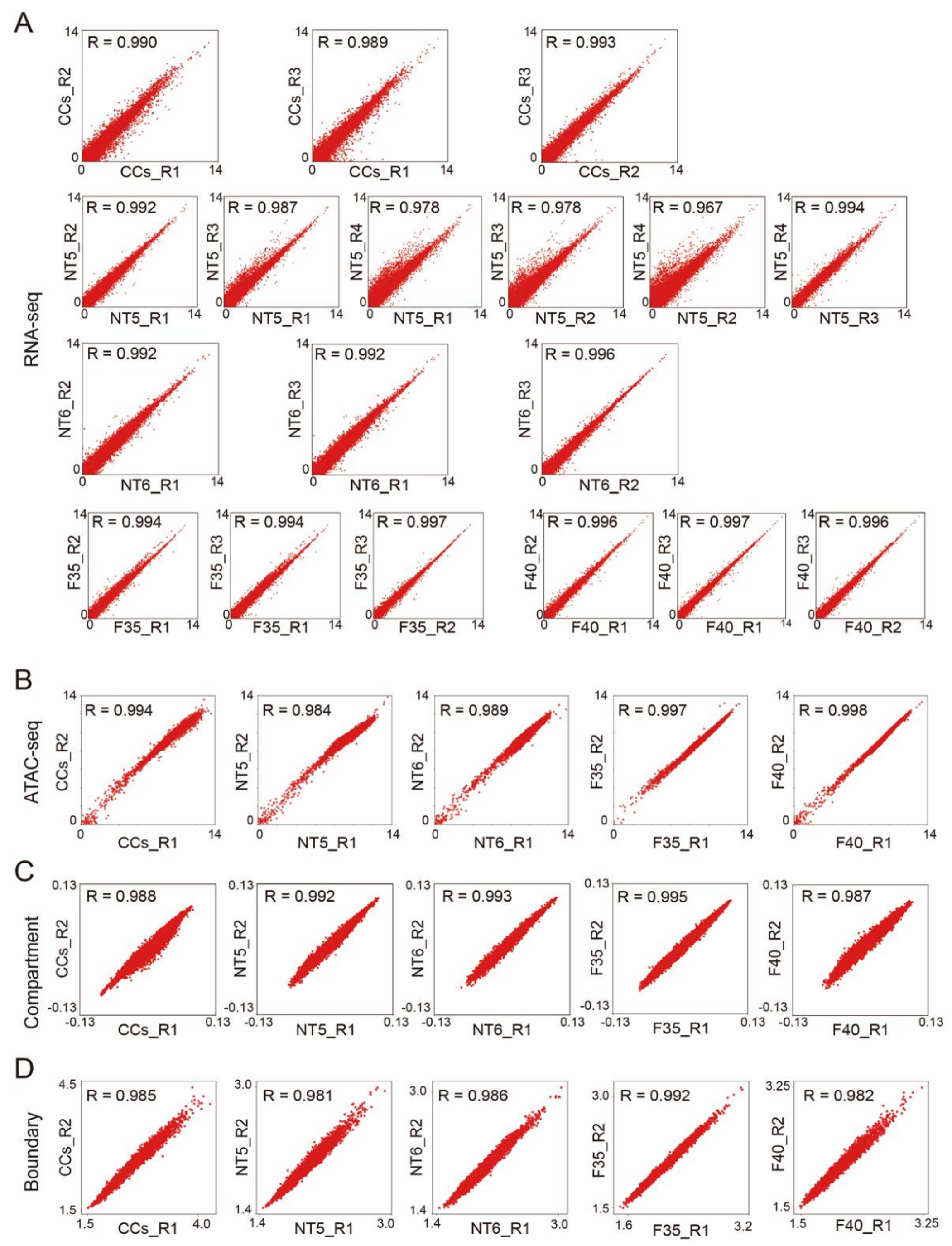

Figure S2. The replicate reproducibility of RNA-seq, ATAC-seq and Hi-C data sets.

A, The Pearson correlation coefficients (PCCs) between two replicates in different RNA-seq data sets. **B,** The PCCs between two replicates in different ATAC-seq data sets. ATAC-seq signals are summed in each 100 Kb region for PCC calculations. **C,** The PCCs calculated from the first principal component (PC1) between two Hi-C replicates. **D,** The PCCs calculated from the boundary insulation scores between two Hi-C replicates.

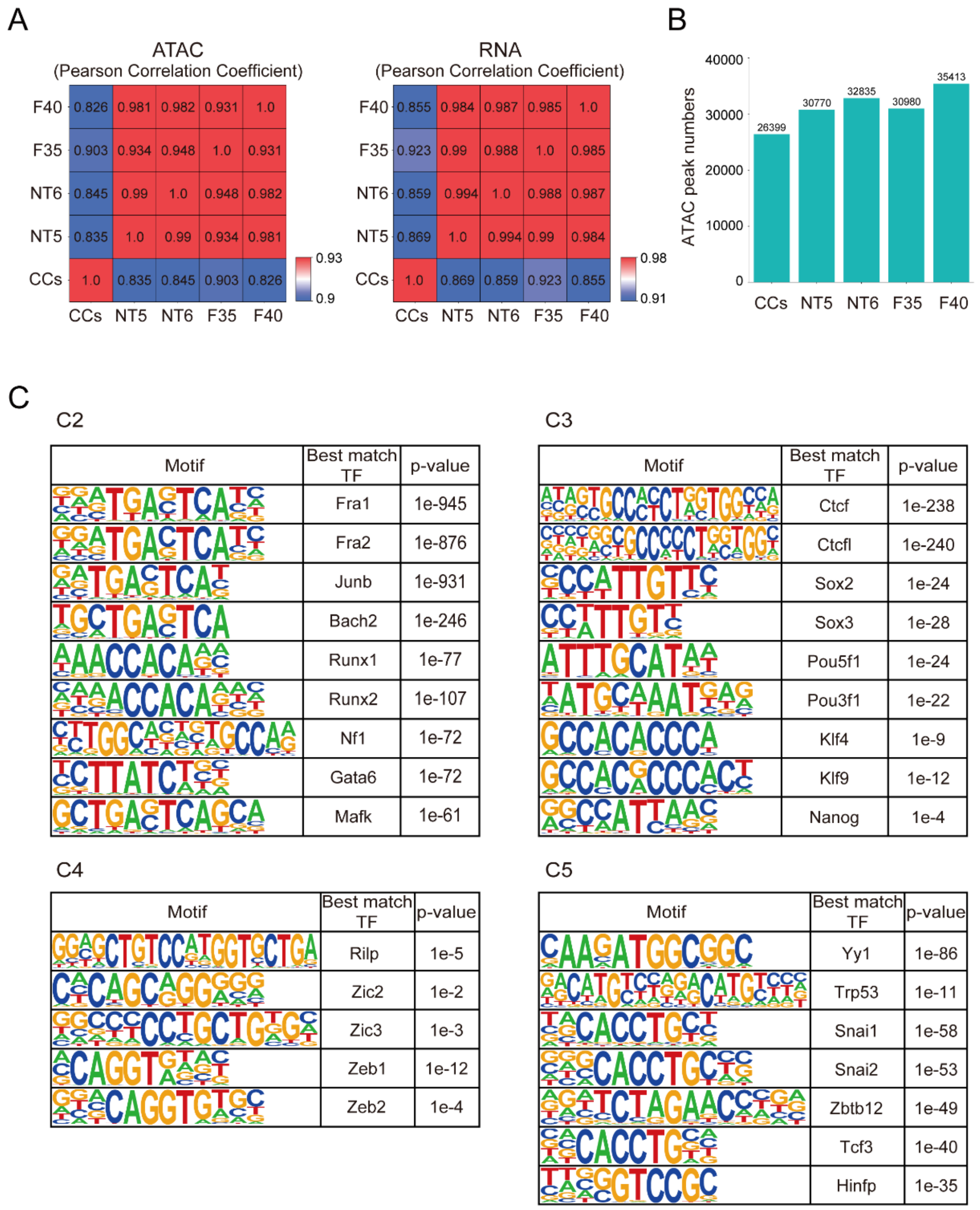

Figure S3. ATAC-seq revealing the global changes of chromatin accessibility in ntESCs.

A, The correlation matrices showing the PCCs among CCs, NT5, NT6, F35 and F40 for ATAC-seq (left) and RNA-seq (right) data, respectively. ATAC-seq signals are summed in each 100 Kb region for PCC calculations, and the gene expression (FPKM) was used for the PCC calculations in RNA-seq data. **B,** Barplot showing the number of ATAC-seq peaks in each cell type (CCs, NT5, NT6, F35 and F40). **C,** The enriched transcription factor (TF) binding motifs in the C2, C3, C4 and C5 peaks.

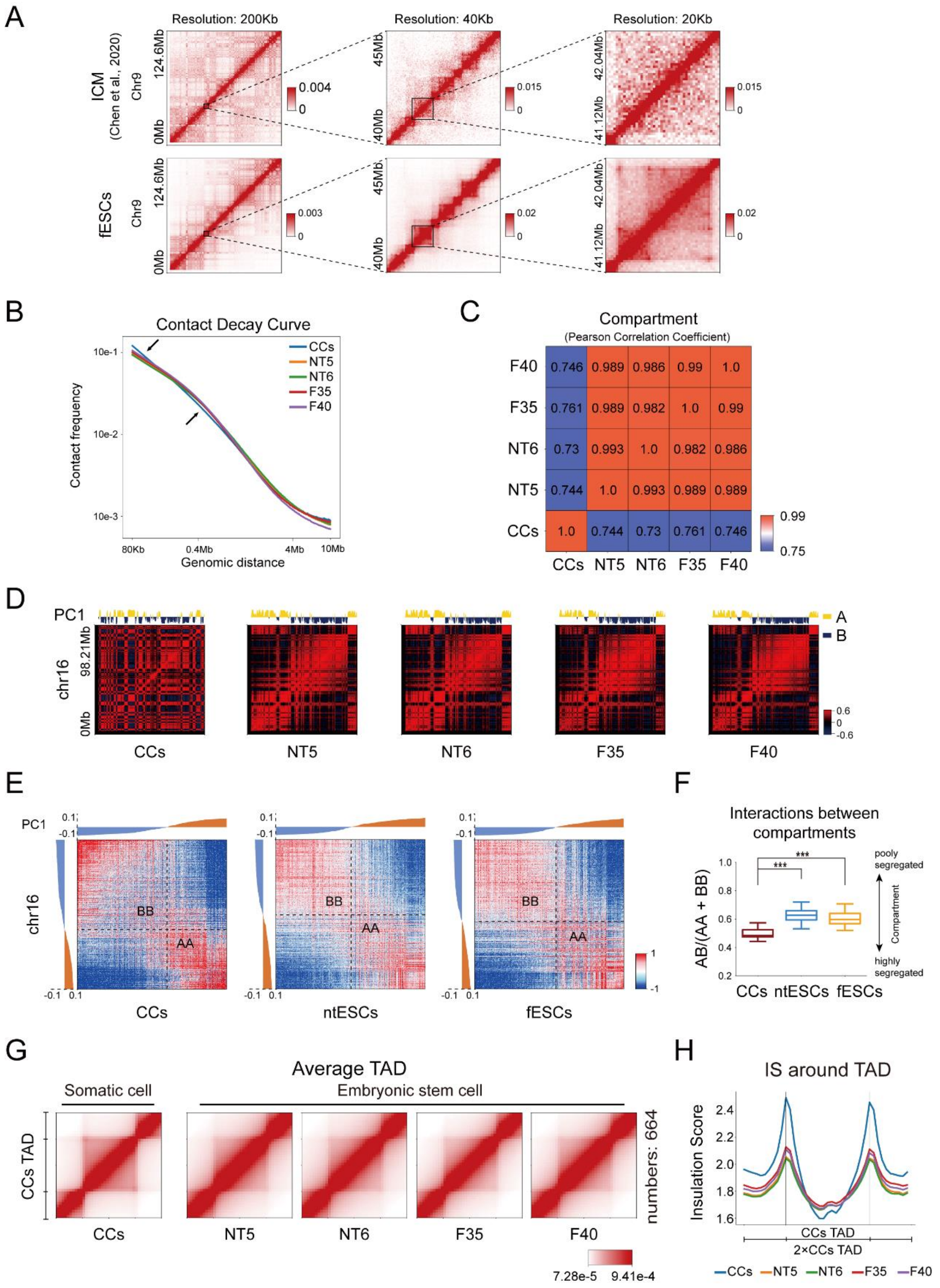

Figure S4. 3D genome transitions in ntESCs.

A, We compared our Hi-C maps of chromosome 9 in fESCs (bottom) to previously published Hi-C maps [1] (top). **B,** The average contact frequencies across the genomic distances in all cell types (blue=CCs, orange=NT5, green=NT6, red=F35 and purple=F40). **C,** The correlation matrix showing the

PCCs of the PC1 values derived from five cell types at the 200 Kb resolutions. **D,** Example of correlation heatmaps. On the top, bar charts show the chromatin compartment states represented by PC1 values. On the bottom, correlation heatmaps of chromosome 16 are shown (200 Kb resolution). **E,** Heatmaps showing the average contact enrichment between pairs of 200-Kb loci arranged by their PC1 values (shown on top and left). The calculation was based on the previous work [2]. **F,** Ratios of interaction frequencies between compartments A & B to those between compartments A & A or B & B. Statistical analysis was performed by using the Wilcoxon ranksum statistic test. *** P <= 0.0005, ** 0.0005 < P value <= 0.005, and * 0.005 < P <= 0.05. The calculation method was based on the published work [1]. **G,** Heatmaps of normalized average interaction frequencies for TADs defined in CCs as well as their nearby regions (+/- 0.5 TAD length) (40 Kb resolution). The TADs with size smaller than 800 Kb are excluded from the calculation. **H,** The average insulation scores of each cell type at the TADs defined in CCs and nearby regions (+/- 0.5 TAD length).

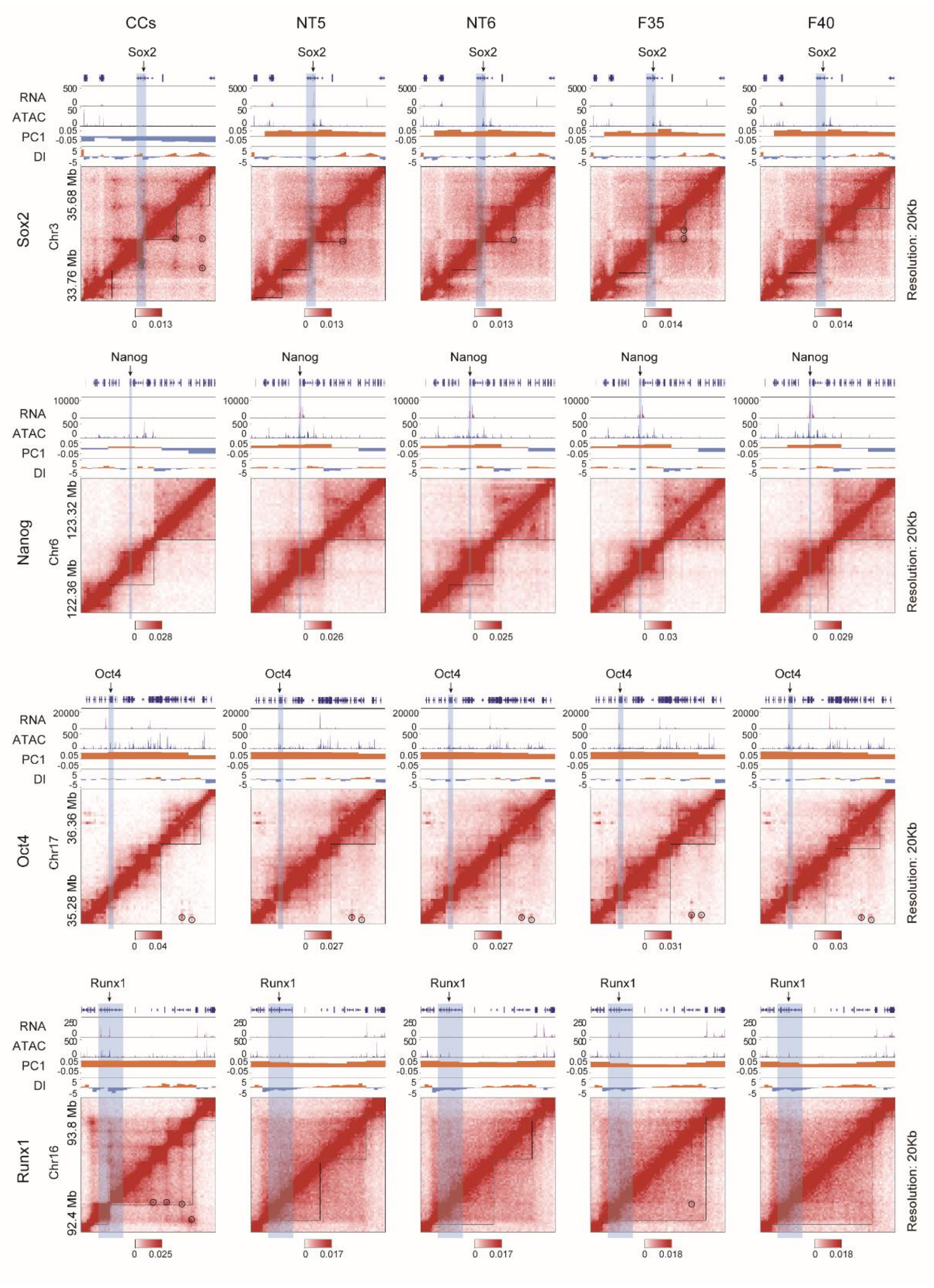

Figure S5. Rewiring of 3D genomes in pluripotent and somatic transcription factors during SCNT reprogramming

Heatmaps show the normalized Hi-C heatmaps (20 Kb resolution), RNA-seq data and ATAC-seq data around the three pluripotent transcription factors (*Sox2*, *Nanog* and *Oct4*) and one somatic transcription factor (Runx1). The PC1 and DI values are also shown to represent chromatin compartments and TADs.

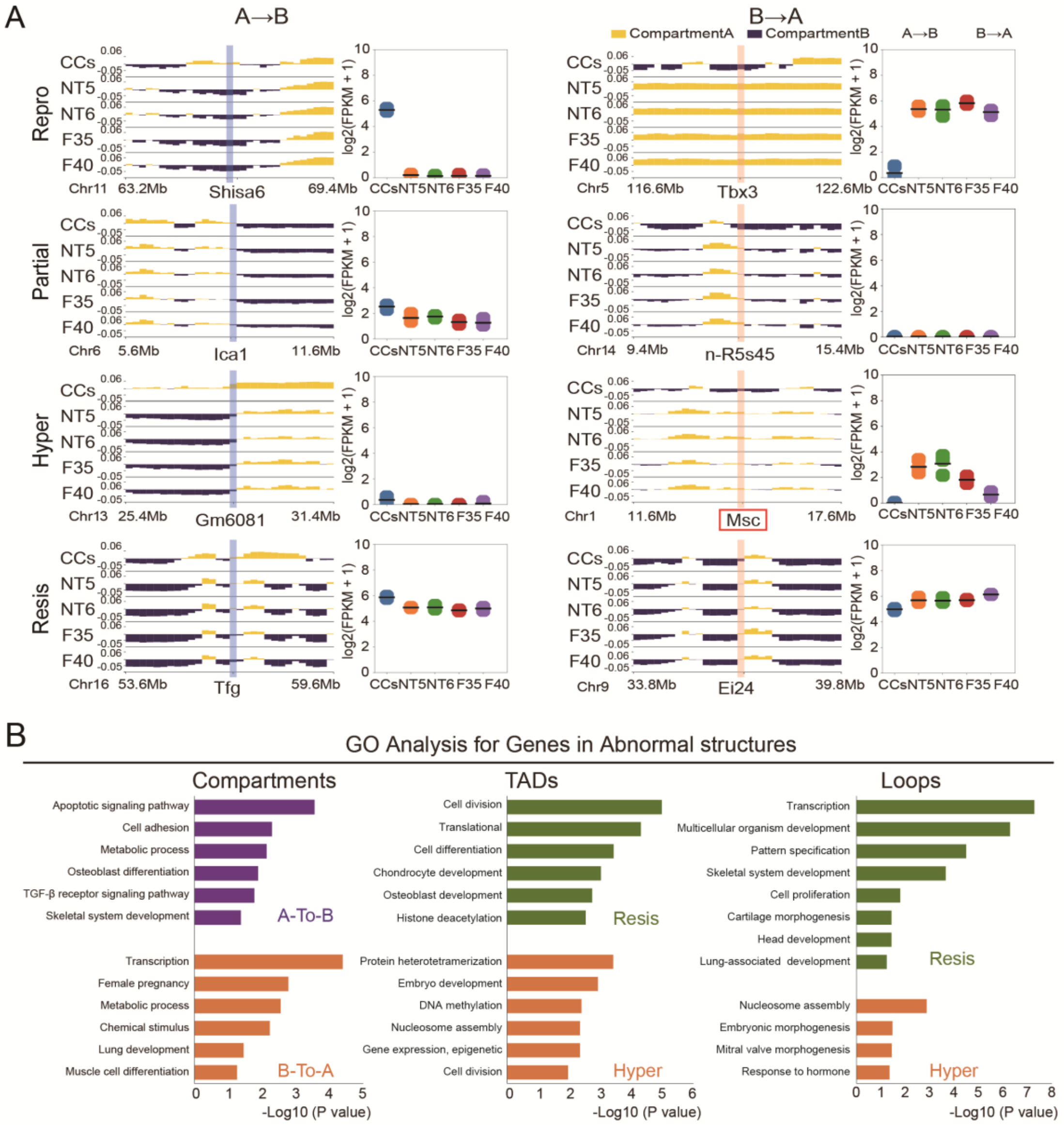

Figure S6. Aberrant chromatin structures in ntESCs.

A, The examples showing different cases of compartmental transitions defined in Figure 4A and the expressions of related genes. Positive PC1 values represent compartment A (yellow), and negative values represent compartment B (dark-blue). The A-to-B and B-to-A compartmental transition between CCs and ESCs (NT5, NT6, F35 and F40) are shown in the light-blue and light-red colors, respectively. **B,** GO analysis of genes related to the abnormal chromatin structures in three layers: Compartment, TAD and Loop.

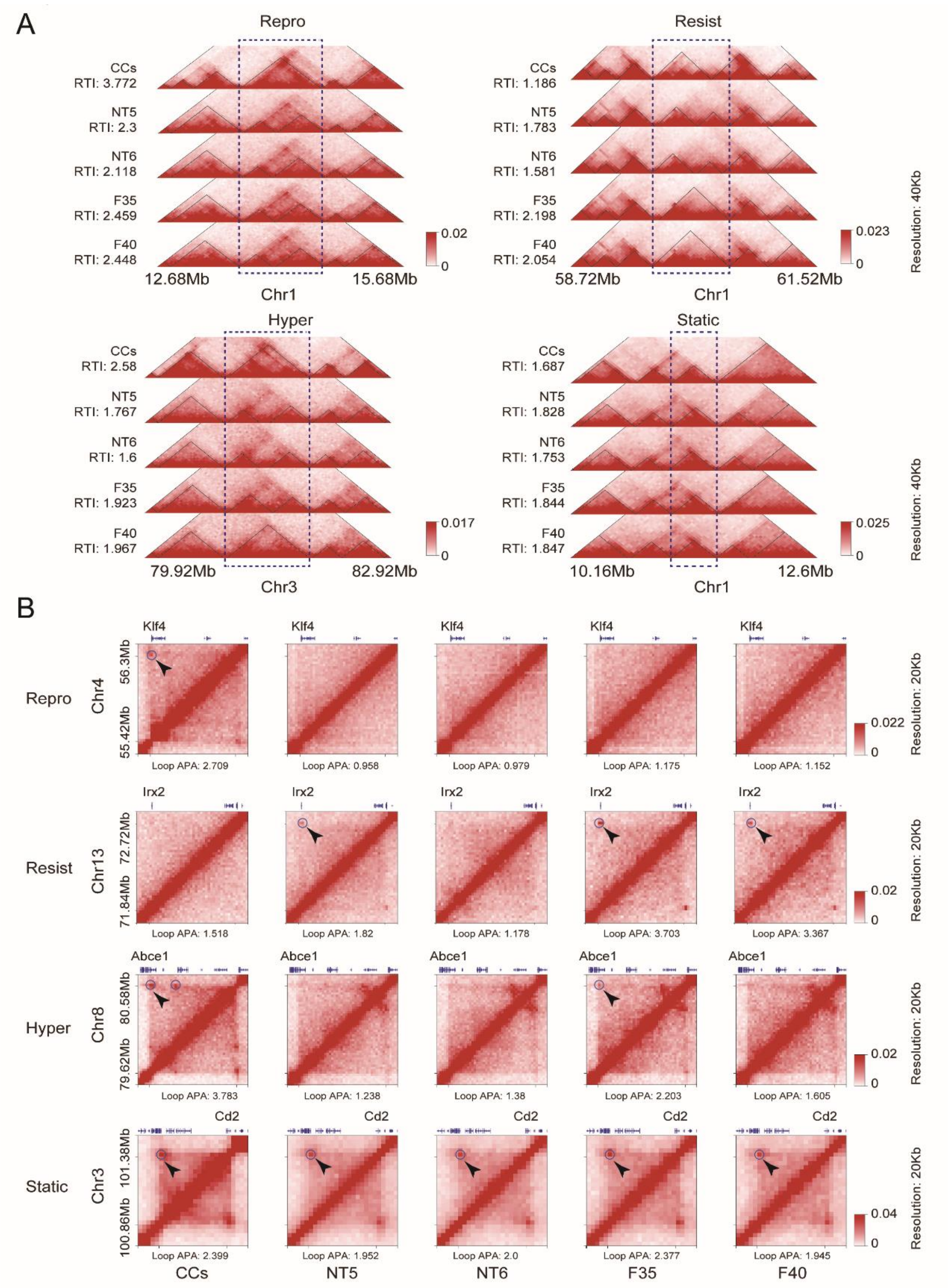

Figure S7. Abnormal TADs and Loops.

A, The examples of 'Repro', 'Resis', 'Hyper' and 'Static' TADs. **B,** The examples of 'Repro', 'Resis', 'Hyper' and 'Static' Loops.

A

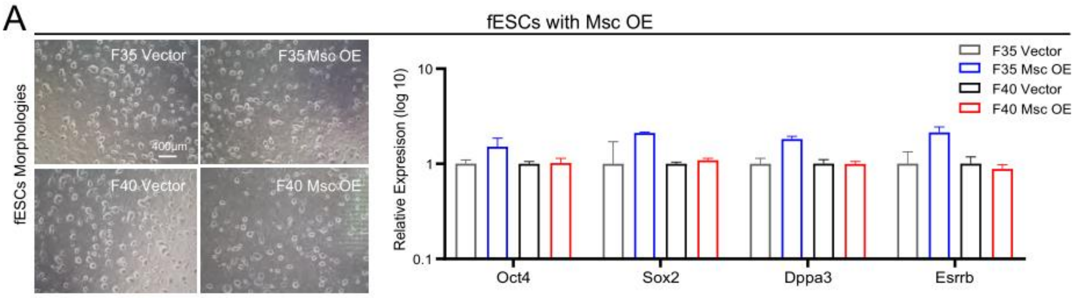

Figure S8. Embryoid body differentiation of ntESCs and fESCs.

A, The morphologies of fESCs with empty *vector* and *MSC* overexpression (OE) (left), scale bar, 400 μm; qRT-PCR analysis of pluripotent genes in empty *vector* and *MSC* OE cell lines, mean ± SEM (n = 3). The Wilcoxon rank-sum test was used in P-value calculations. * 0.01 <= P < 0.05, ** 0.001 <= P < 0.01 and *** P < 0.001.

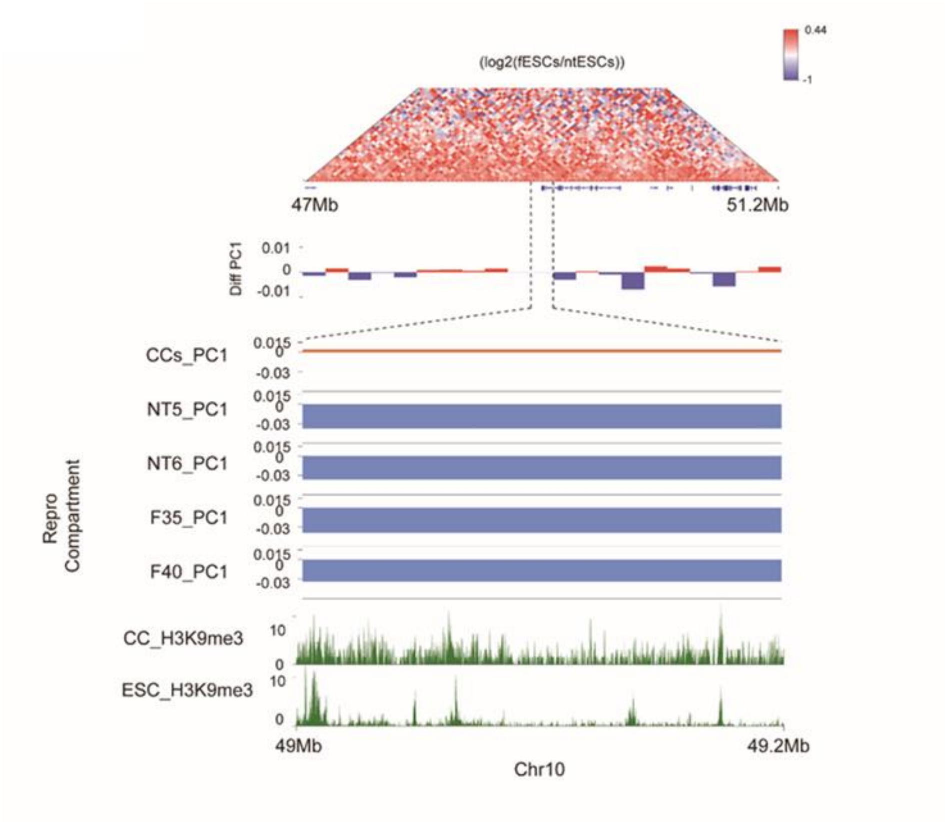

Figure S9. An example of the Reprogrammed compartment region.

The differential interaction frequencies (log2(fESCs/ntESCs)) and corresponding H3K9me3 signals around the A-to-B reprogrammed compartment region (Chr10: 49 Mb – 49.2Mb). The bottom tracks show the H3K9me3 signal in CCs [1] and ESC [3] respectively within the selected region.

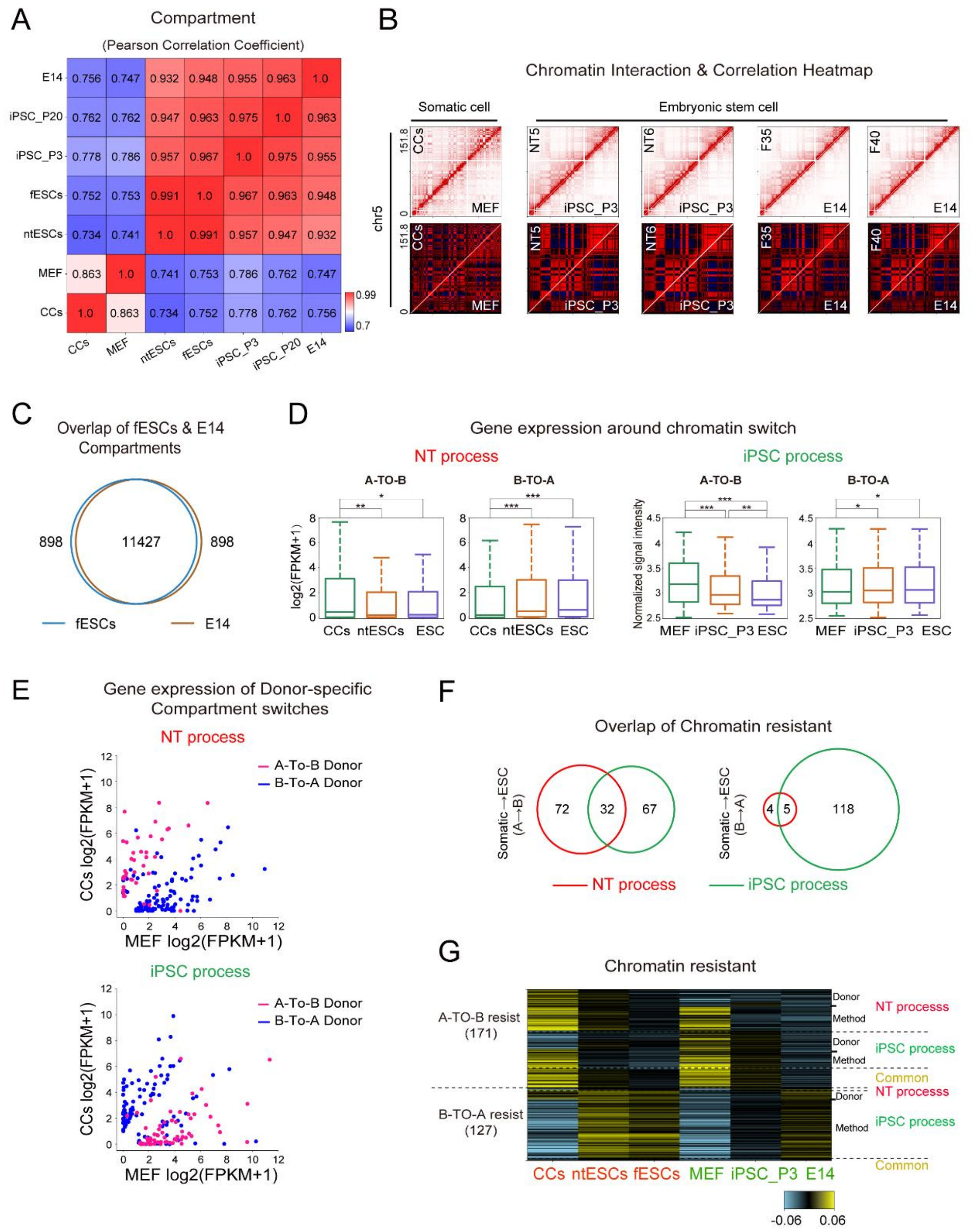

Figure S10. Comparison of 3D structures between ntESCs and iPSCs.

A, The PCCs of PC1 derived from different cell types (200 Kb resolution). **B,** The top and bottom heatmaps showing the normalized Hi-C interaction frequencies and corresponding correlation matrices for chromosome 5 (200 Kb resolution) in the cell types of NT process (CCs, NT5, NT6, F35 and F40) (top left in all subfigures) and iPSC process (MEF, iPSCs and E14) (bottom right in all subfigures). **C,** The Venn diagrams showing the overlap of compartmental states between fESCs and E14. The numbers are also shown. **D,** Boxplots

showing the average gene expressions in the compartment switch regions in both NT and iPSC processes. Statistical analysis was performed by using the Wilcoxon rank-sum test. *** P <= 0.0005, ** 0.0005 < P <= 0.005, and * 0.005 < P <= 0.05. **E,** The scatterplots showing the differential expression of compartment-related genes derived from different donor somatic cells in CCs and MEF. ‘A-To-B Donor’ and ‘B-To-A Donor’ represent the genes related to the NT-specific and iPSC-specific compartment switches defined in the Figure 6D. The x-axis and y-axis represent the gene expression of MEF and CCs (log2(FPKM+1)) respectively. **F,** The Venn diagrams showing the overlap of resistant compartment regions between NT and iPSC processes. The numbers are also shown. **G,** The heatmap showing the PC1 values of resistant compartments. The number of A -to-B or B-to-A resistant compartments in the NT and iPSC processes are shown on the left side of the heatmap, respectively. Donor-specific, method-specific and common resistant compartments of the NT and iPSC processes are shown on the right side of the heatmaps, respectively.

Supplemental Methods

Karyotype analysis

The cells were incubated in ESCs medium with 0.4 μg/ml colcemid for 2 hr and harvested from MEFs with 0.05% Trypsin-EDTA. After incubation in hypotonic solution with 0.075 M KCl at 37 °C for 20 min and centrifuge, the cells were fixed with a methanol/acetic acid mixture (3:1, v/v) and centrifuge, and repeat twice. The fixed cells were mounted on glass slides (precooling in advance at 4 °C) and stained with Giemsa for 10 min after drying. The numbers of metaphase chromosomes were counted.

Alkaline phosphatase (AP) staining

AP staining was performed with a BCIP/NBT Alkaline Phosphatase Color Development Kit according to the manufacturer's instructions. Cells were fixed with 4 % paraformaldehyde for 1-2 min followed by rinsing with 1X TBS-T buffer (20 mM Tris-HCl, pH 7.4, 150 mM NaCl, and 0.05 % Tween-20). Stain solution was applied to cover the cells at room temperature in dark for 30 min. After rinsing the cells with 1 X TBS-T buffer, cell images were taken.

RNA extraction and quantitative RT-PCR

Trizol was used to extract total RNA following manufacturer's instruction. Then total RNA was treated with DNase before reverse transcription using the M-MLV reverse transcriptase system (Promega). For PCR, 1 μl cDNA was used as the template of 25 μl reaction system by using SYBP qPCR Master Mix (Vazyne, Q711) and relative gene expression was calculated using the ΔCt method. The primer sequences are listed in Table S2.

Immunofluorescence, confocal microscopy

ES cells grew on the gelatin coated cover slides were fixed in 4% paraformaldehyde. After permeabilization and blocking treatment, the cells were incubated with the first antibody at a dilution of 1:400 (Oct4, Abcam AB181557; Nanog, Abcam ab80892 and SSEA-1, Abcam ab16285). The secondary antibody was incubated with the samples after three times of washing. DNA was labeled by DAPI. Stained cells mounted on slides were observed by using a Zeiss confocal microscope (Zeiss LSM 800).

Chimera Construction

To produce chimeric mice, about 10–15 ES cells were microinjected into the ICR blastocysts using piezo-actuated microinjection pipette. After culture for 2-3 hr, the re-expanded blastocysts were transplanted into uteri of pseudo-pregnant mice. And chimeric mice were identified by coat color.

Generation of *Msc* overexpression fESCs

Msc cDNA was amplified from mouse ovary cDNA and cloned into the plvx-efla-IREs-hygroB vector using the BamH I and EcoR I sites. fESCs were nucleofected with the plvx-efla-IREs-hygroB vector as control and plvx-efla-IREs-*Msc*-hygroB plasmid as *Msc* overexpression. Then, fESCs were stably selected in the presence of 500 μg/mL hygroB. For induction of EB differentiation, fESCs with *Msc* OE and empty vector were cultured in the absence of LIF on low-attachment dishes to induce EB differentiation. Day 5 and Day 10 EBs were collected for qRT-PCR and primer sequences of three layers are listed in Table S2.

RNA sequencing and data processing

Total RNA was extracted from the different cell types using TRIzol (Invitrogen, Thermo Fisher Scientific). Libraries were constructed and sequenced by Annoroad Gene Technology. The raw RNA-seq data were trimmed 15 bp using Trimmomatic (version 0.36) [4]. The trimmed reads were aligned to mouse reference genome (mm10) using TopHat2 (version2.1.1) [5], and the gene expression values (FPKM) were calculated using Stringtie (version 2.1.4) [6]. The logarithm of gene expression (log2(FPKM+1)) for each technical replicate was used to calculate Pearson correlation coefficients (PCCs) among RNA-seq libraries. Differentially expressed genes between any two cell types were identified using DESeq2 (version 1.30.1) [7] with adjusted p value smaller than 0.05. The GO analysis was performed with DAVID (version 6.8) [8]. HTSeq (version 0.6.1p1) [9] was used to calculate gene counts from replicate experiments.

ATAC sequencing and data processing

ATAC-seq was performed as previously described [10]. In brief, a total of 50,000 cells were washed once with cold PBS and re-suspended in lysis buffer (10 mM Tris-HCl, pH 7.4, 10 mM NaCl, 3 mM $MgCl_2$ and 0.1% IGEPAL CA-630). The suspension of nuclei was then centrifuged at 4℃, followed by the addition of transposition reaction mix of TruePrep DNA Library Prep Kit V2 for Illumina (Vazyme, TD501). Samples were then PCR amplified and incubated at 37℃ for 30 min. DNA was isolated using a Qiagen MinElute Kit. ATAC-seq libraries were subjected to 9 cycles for amplification. Libraries were purified with a Qiagen PCR Cleanup Kit. Library concentration was measured using a KAPA Library Quantification kit (KK4824) according to the manufacturer's instructions. Library integrity was checked by gel electrophoresis. Finally, the ATAC libraries were sequenced on Illumina Hiseq X-Ten.

Raw reads were trimmed 15 bp using Trimmomatic (version 0.36) [4], and then aligned to mm10 using Bowtie2 (version 2.3.5.1) [11] with default parameters. Only the uniquely mapped reads were preserved. The RPKM was calculated at 100 bp resolution using deeptools [12] and the logarithm of the summation of RPKMs in 100 kilobases was used to calculate PCCs among ATAC libraries. The peaks were called using MACS2 (version 2.1.2) [13] with 0.01 as FDR threshold, and the reproducible peaks were selected using irreproducible discovery rate (IDR) [14] as a measurement. The IDR threshold was set to be 0.01 for cell types CCs, NT5, NT6 and F35. Due to better experiment quality in reproducibility, the IDR threshold was set to be 0.001 for cell type F40 to balance the number of called peaks.

In ATAC-seq peak analysis, if two peaks overlapped in genomic positions, these two peaks were considered to be aligned peak, and the merged genomic positions of the two peaks were used for clustering and motif analysis. The ATAC-seq peaks were divided into three clusters (C1, C2, C3) using K-means according to the peak intensity. To find the differences between ntESCs and fESCs, the C3 peaks with intensity difference between ntESCs and fESCs were further identified using Diffbind (https://anaconda.org/bioconda/bioconductor-diffbind) (version 2.14.0) with 0.05 as the threshold for p value. The peaks with significantly higher intensities in fESCs were defined as C4, and the peaks with reserve intensity trend were defined as C5. The ChIPseeker (version 1.26.2) [15] was used to annotate each cluster of ATAC peaks. The software GREAT (version 4.0.4) [16] was used to find the target genes for regulatory elements. Then DAVID (version 6.8) [8] was used to perform GO analysis on these genes. The motif enrichments for candidate peaks were scanned using homer (version 4.11.1) [17] with default parameters.

Hi-C sequencing and data processing

The *in situ* Hi-C libraries were generated by following the previous protocol [18] and were sequenced on IIlumina HiSeq X Ten. The Hi-C data were aligned, processed and corrected using HiCHap (version 1.6) [19]. Briefly, the two ends of paired reads were mapped to mm10 using bowtie2 [11] with default parameters, and the noise filtering was performed by following the previous process, including removing duplicates, self-ligations, dangling reads and the paired reads with same direction [20]. The iterative correction was used to correct biases in Hi-C data [20]. The contact frequencies and genomic distances were transformed by using log10 function when plotting their relationship. The interaction matrix of ntESCs was calculated from merged data sets of NT5 and

NT6, and interaction matrix of fESCs was calculated from merged data sets of F35 and F40. Generally, the compartments, topologically associating domains (TADs) and chromatin loops were identified at 200 Kb, 40 Kb and 20 Kb resolutions respectively without explicit statement. However, the chromosome X was excluded from these calculations due to the absence of obvious structures. The 5 Kb resolution was used in ntESCs and fESCs when identifying high-resolution chromatin loops.

Compartment identification and analysis

The principal component analysis (PCA) was used to calculate chromosomal compartments at 200 Kb resolution. The first principal component (PC1) was used to identify the chromosomal compartments, in which the ATAC-seq data were used to determine the A (active) and B (inactive) compartments. The compartment similarity was defined as the PCCs between two PC1 values. In order to further analyze the dynamic changes between cell types, compartments were divided into 8 categories based on the value of PC1. For the compartment undergoing A-to-B and B-to-A switches from CCs to fESCs, if the fold change between PC1 value of ntESCs and PC1 value of fESCs was greater than 0.2 and less than 1.5, this compartment was defined as ‘Repro’ (Reprogrammed). These A-to-B and B-to-A switches were labeled as ABrB and BArA respectively. If the fold change was less than 0.2, the switched compartment was defined as ‘Partial’, which was labeled as ABpB and BApA, respectively. If the fold change was greater than 1.5, the switched compartment was defined as ‘Hyper’, which was labeled as ABhB and BAhA, respectively. The denotation ‘Resis’ (Resistant) referred to the compartments which were active in CCs and ntESCs but inactive in fESCs (labeled as AAB), and the compartments which were inactive in CCs and ntESCs but active in fESCs (labeled as BBA). In the gene expression analysis, if a gene occupied two or more compartmental bins, it was assigned to the compartmental bin where the center of this gene located. The compartment differences between two cell types were calculated by subtracting the corresponding two PC1 values.

TAD identification and analysis

TADs were called at 40 Kb resolution by using the directionality index (DI) based hidden markov model (HMM) implemented in HiTAD software (version 0.4.2-r1) [21], in which the fixed 400 Kb window was used and the bottom boundaries reproducible between two replicates were preserved. The boundaries between two cell types were aligned using HiTAD. If the boundary in one cell type was not aligned to any boundary in the other cell type, the exact

position of this boundary was used to calculate the insulation score for the two cell types. The boundary insulation score was defined as the summation of inter-domain and intra-domain interactions divided by the inter-domain interactions [22] using the bias-corrected matrix. The PCC between two sets of insulation score was used to measure the boundary-level similarity between two Hi-C libraries.

For the boundary presentation, the aligned TAD boundaries derived from different cell types were combined together. The left and right 600 Kb regions of combined boundaries were used to calculate the average interaction frequencies of each cell type, in which the interaction frequencies were normalized by dividing the summation of interaction matrix. For the TAD presentation, the TADs (defined in CCs) were rescaled to the 800 Kb interval (~average length of TADs) by using python packages from the previously published work (https://github.com/rysterzhu/Chromatin-Structure-in-SCNT). We further normalized the matrix of interaction frequencies by dividing the summation of the matrix. The upstream and downstream 400 Kb chromatin regions of left and right boundaries were also calculated for TAD presentation.

In order to further analyze the dynamic changes among cell types, the relative TAD intensity (RTI) [23] was used to divide the TADs into 4 categories following the method of a previous study [24]. Specifically, the TADs with the RTI scores ($-0.2 < RTI_{(CCs)} - RTI_{(fESCs)} < 0.2$) were considered as 'Static'. In the rest TADs, if one TAD disappears or appears in fESCs compared to CCs, this TAD was defined as dynamic TAD. In all dynamic TADs, if the RTI scores of ntESCs were closer to those of fESCs ($0.7 < (RTI_{(ntESCs)}-RTI_{(CCs)})/(RTI_{(fESCs)}-RTI_{(CCs)}) < 1.2$), these TADs were defined as 'Repro' (Reprogrammed). If the RTI scores of ntESCs were closer to those of CCs ($(RTI_{(ntESCs)}-RTI_{(CCs)})/(RTI_{(fESCs)}-RTI_{(CCs)}) < 0.7$), they were defined as 'Resis' (Resistant). If the RTI scores were far away from the $RTI_{(CCs)}$ ($(RTI_{(ntESCs)}-RTI_{(CCs)})/(RTI_{(fESCs)}-RTI_{(CCs)}) > 1.2$), they were defined as 'Hyper'. If the genomic distance between gene center and one of the two TAD boundaries was less than 40 Kb, this gene was considered to be involved in this TAD in gene analysis.

Chromatin loop identification and analysis

The chromatin loops were called at 20 Kb resolution by using a previous pipeline [25]. Briefly, in the first round, HiCCUPS algorithm [18] was used to detect locally significant chromatin interactions using 0.05 as p value threshold and these initially called chromatin interactions were grouped according to Euclidean distance. Then the most significant interaction in each group was selected as representative in the second round of grouping. Finally, the most

significant interaction in each second-round group was selected as chromatin loop and the corresponding q-value was calculated. In this work, the chromatin loops with q-value smaller than 10^{-4} and interaction frequency larger than 50 were selected in the five cell types (CCs, NT5, NT6, F35 and F40). The corresponding thresholds were set to be 10^{-6} and 60, respectively in the two merged types (ntESCs and fESCs).

When aligning chromatin loops among three cell types (CCs, ntESCs and fESCs) in loop analysis, all chromatin loops with Euclidean distance smaller or equal to $\sqrt{2}$ were grouped. If Euclidean distance between a chromatin loop to the geometric center of the group was also smaller or equal to $\sqrt{2}$, this chromatin loop was considered to be aligned to this group. The located bin of group geometric center was used in the chromatin loop strength analysis. The loop strength was calculated using the Aggregate Peak Analysis (APA) proposed in previous work [18]. Then the aligned chromatin loops were divided into 4 categories in a similar way as aforementioned TAD classifications. Briefly, the chromatin loops with the APA scores ($-0.2 < APA_{(CCs)} – APA_{(fESCs)} < 0.2$) were considered as ‘Static’. In the rest of the chromatin loops, the dynamic loop was the one with disappearance or appearance in fESCs compared to CCs. The loop classifications for ‘Repro’, ‘Resis’ and ‘Hyper’ were defined in the same way as those in TADs with the same parameters, except that the APA score was used here. If the genomic distance between the gene center and one of the loop anchors was less than 20 Kb, this gene was considered to be involved in this loop in gene analysis.

Identification of H3K9me3-marked compartments and TADs

The definition of H3K9me3-marked compartment and TAD was derived from previously published work [1] with some modifications. In this study, compartmental bins with H3K9me3 signals higher than the 1.4 times input signals were defined as H3K9me3-marked compartments, in which the RPKM normalization was applied to ChIP-seq signals using deeptools [12] with default parameters. Then 2836 compartment bins in CCs were marked with H3K9me3. Similarly, TADs with H3K9me3 signals higher than the 1.4 times input signals were defined as H3K9me3-marked TADs. In total, 224 CC-specific TADs and 22 fESC-specific TADs were marked with H3K9me3.

References

1. Chen M, Zhu Q, Li C, Kou X, Zhao Y, Li Y*, et al.* Chromatin architecture reorganization in murine somatic cell nuclear transfer embryos. *Nat Commun* 2020, **11**(1)**:** 1813.
2. Bonev B, Mendelson Cohen N, Szabo Q, Fritsch L, Papadopoulos GL, Lubling Y*, et al.* Multiscale 3D Genome Rewiring during Mouse Neural Development. *Cell* 2017, **171**(3)**:** 557-572 e524.
3. Chronis C, Fiziev P, Papp B, Butz S, Bonora G, Sabri S*, et al.* Cooperative Binding of Transcription Factors Orchestrates Reprogramming. *Cell* 2017, **168**(3)**:** 442-459 e420.
4. Bolger AM, Lohse M, Usadel B. Trimmomatic: a flexible trimmer for Illumina sequence data. *Bioinformatics* 2014, **30**(15)**:** 2114-2120.
5. Trapnell C, Pachter L, Salzberg SL. TopHat: discovering splice junctions with RNA-Seq. *Bioinformatics* 2009, **25**(9)**:** 1105-1111.
6. Pertea M, Pertea GM, Antonescu CM, Chang TC, Mendell JT, Salzberg SL. StringTie enables improved reconstruction of a transcriptome from RNA-seq reads. *Nat Biotechnol* 2015, **33**(3)**:** 290-295.
7. Love MI, Huber W, Anders S. Moderated estimation of fold change and dispersion for RNA-seq data with DESeq2. *Genome Biol* 2014, **15**(12)**:** 550.
8. Jiao X, Sherman BT, Huang da W, Stephens R, Baseler MW, Lane HC*, et al.* DAVID-WS: a stateful web service to facilitate gene/protein list analysis. *Bioinformatics* 2012, **28**(13)**:** 1805-1806.
9. Anders S, Pyl PT, Huber W. HTSeq--a Python framework to work with high-throughput sequencing data. *Bioinformatics* 2015, **31**(2)**:** 166-169.
10. Buenrostro JD, Wu B, Chang HY, Greenleaf WJ. ATAC-seq: A Method for Assaying Chromatin Accessibility Genome-Wide. *Current protocols in molecular biology* 2015, **109:** 21 29 21-21 29 29.
11. Langmead B, Salzberg SL. Fast gapped-read alignment with Bowtie 2. *Nature methods* 2012, **9**(4)**:** 357-359.
12. Ramirez F, Ryan DP, Gruning B, Bhardwaj V, Kilpert F, Richter AS*, et al.* deepTools2: a next generation web server for deep-sequencing data analysis. *Nucleic acids research* 2016, **44**(W1)**:** W160-165.
13. Zhang Y, Liu T, Meyer CA, Eeckhoute J, Johnson DS, Bernstein BE*, et al.* Model-based analysis of ChIP-Seq (MACS). *Genome Biol* 2008, **9**(9)**:** R137.
14. Li QH, Brown JB, Huang HY, Bickel PJ. Measuring Reproducibility of High-Throughput Experiments. *Ann Appl Stat* 2011, **5**(3)**:** 1752-1779.
15. Yu G, Wang LG, He QY. ChIPseeker: an R/Bioconductor package for ChIP peak annotation, comparison and visualization. *Bioinformatics* 2015, **31**(14)**:** 2382-2383.
16. McLean CY, Bristor D, Hiller M, Clarke SL, Schaar BT, Lowe CB*, et al.* GREAT improves functional interpretation of cis-regulatory regions. *Nature biotechnology* 2010, **28**(5)**:** 495-501.
17. Heinz S, Benner C, Spann N, Bertolino E, Lin YC, Laslo P*, et al.* Simple combinations of lineage-determining transcription factors prime cis-regulatory elements required for macrophage and B cell identities. *Molecular cell* 2010, **38**(4)**:** 576-589.
18. Rao SS, Huntley MH, Durand NC, Stamenova EK, Bochkov ID, Robinson JT*, et al.* A 3D map of the human genome at kilobase resolution reveals principles of chromatin looping. *Cell* 2014, **159**(7)**:** 1665-1680.
19. Luo H, Li X, Fu H, Peng C. HiCHap: a package to correct and analyze the diploid Hi-C data. *BMC Genomics* 2020, **21**(1)**:** 746.

20. Imakaev M, Fudenberg G, McCord RP, Naumova N, Goloborodko A, Lajoie BR*, et al.* Iterative correction of Hi-C data reveals hallmarks of chromosome organization. *Nature methods* 2012, **9**(10)**:** 999-1003.

21. Wang XT, Cui W, Peng C. HiTAD: detecting the structural and functional hierarchies of topologically associating domains from chromatin interactions. *Nucleic Acids Res* 2017, **45**(19)**:** e163.

22. Nagano T, Lubling Y, Varnai C, Dudley C, Leung W, Baran Y*, et al.* Cell-cycle dynamics of chromosomal organization at single-cell resolution. *Nature* 2017, **547**(7661)**:** 61-67.

23. Ke Y, Xu Y, Chen X, Feng S, Liu Z, Sun Y*, et al.* 3D Chromatin Structures of Mature Gametes and Structural Reprogramming during Mammalian Embryogenesis. *Cell* 2017, **170**(2)**:** 367-381 e320.

24. Zhang K, Wu DY, Zheng H, Wang Y, Sun QR, Liu X*, et al.* Analysis of Genome Architecture during SCNT Reveals a Role of Cohesin in Impeding Minor ZGA. *Molecular cell* 2020, **79**(2)**:** 234-250 e239.

25. Wen Z, Huang ZT, Zhang R, Peng C. ZNF143 is a regulator of chromatin loop. *Cell biology and toxicology* 2018, **34**(6)**:** 471-478.
